# Critical timing and extent of public health interventions to control outbreaks dominated by SARS-CoV-2 variants in Australia: a mathematical modelling study

**DOI:** 10.1016/j.ijid.2021.11.024

**Published:** 2022-02

**Authors:** Zhuoru Zou, Christopher K. Fairley, Mingwang Shen, Nick Scott, Xianglong Xu, Zengbin Li, Rui Li, Guihua Zhuang, Lei Zhang

**Affiliations:** a China–Australia Joint Research Centre for Infectious Diseases, School of Public Health, Xi'an Jiaotong University Health Science Centre, Xi'an, 710061, China; b Melbourne Sexual Health Centre, Alfred Health, Melbourne, VIC 3053, Australia; c Central Clinical School, Faculty of Medicine, Monash University, Melbourne, VIC 3800, Australia; d Burnet Institute, Melbourne, VIC 3004, Australia

**Keywords:** COVID-19, SARS-CoV-2, Variants, Public health interventions, Vaccination, Modelling

## Abstract

**Objectives:**

The exact characteristics of a coronavirus disease 2019 (COVID-19) outbreak that trigger public health interventions are poorly defined. The aim of this study was to assess the critical timing and extent of public health interventions to contain COVID-19 outbreaks in Australia.

**Methods:**

A practical model was developed using existing epidemic data in Australia. The effective combinations of public health interventions and the critical number of daily cases for intervention commencement under various scenarios of changes in transmissibility of new variants and vaccination coverage were quantified.

**Results:**

In the past COVID-19 outbreaks in four Australian states, the number of reported cases on the day that interventions commenced strongly predicted the size and duration of the outbreaks. In the early phase of an outbreak, containing a wildtype-dominant epidemic to a low level (≤10 cases/day) would require effective combinations of social distancing and face mask use interventions to be commenced before the number of daily reported cases reaches six. Containing an Alpha-dominant epidemic would require more stringent interventions that commence earlier. For the Delta variant, public health interventions alone would not contain the epidemic unless the vaccination coverage was ≥70%.

**Conclusions:**

This study highlights the importance of early and decisive action in the initial phase of an outbreak. Vaccination is essential for containing variants.

## Highlights

• Social distancing and face mask use could reduce the effective reproduction number (R_e_) to 1 in wildtype epidemics.
• Containing wildtype epidemics required interventions to begin before the number of daily reported cases was >6.
• Containing Alpha epidemics required more stringent interventions that commenced earlier.
• Containing Delta epidemics required 70% vaccination combined with other interventions.
• Increasing vaccination coverage will allow less strict interventions and increase the tolerance for the timing of intervention commencement.

## 1 Introduction

The coronavirus disease 2019 (COVID-19) pandemic continues to cause a catastrophic health and economic crisis around the world (McKee and Stuckler, 2020; World Health Organization, 2021). To prevent the consequences of the COVID-19 epidemic, 22 vaccine candidates have been approved by the World Health Organization (Craven, 2021). Yet, achieving global herd immunity with these vaccines will take time, given the existing disparity in COVID-19 vaccination across the globe (Forman et al., 2021). Non-pharmaceutical interventions remain the most effective means for COVID-19 control until herd immunity can be achieved. Non-pharmaceutical interventions have been successful in controlling the wildtype-dominant outbreaks in countries such as Australia, China, New Zealand, and Singapore in the past. These past experiences have demonstrated that early intervention results in more effective control of outbreaks.

However, the emergence of severe acute respiratory syndrome coronavirus 2 (SARS-CoV-2) variants with much stronger transmissibility has substantially changed the thresholds for public health interventions. For instance, the Alpha-dominant epidemic in the UK in late 2020 forced the government to elevate lockdown restrictions from tier 3 to tier 4 in order to combat the epidemic surge (Kirby, 2021). In the most recent epidemics dominated by the Delta variant, two of the most populous Australian states, New South Wales and Victoria, failed to revert the epidemic trend to achieve their original ‘zero community transmission’ target and resorted to policies of ‘living with COVID-19’ (Jose and Barrett, 2021). In comparison, China and New Zealand have managed to contain the Delta-dominant epidemics to a very low level, despite reports of sporadic cases. An understanding of the timeliness and extent of public health interventions, taking into consideration population vaccination coverage and variant transmissibility, would provide important evidence to explain the differences in COVID-19 control and epidemic severity in these countries.

Outbreak surveillance for early COVID-19 detection is essential to inform control measures. It enables stakeholders to commence interventions in time to avoid lengthy and extensive restrictions down the track and minimize health and economic losses. Several studies have explored surveillance indicators for the early detection of COVID-19 outbreaks, such as COVID-19-related digital data streams and SARS-CoV-2 viral fragment detection in wastewater (Güemes et al., 2021; Hasan et al., 2021; Kogan et al., 2021). In addition, modelling studies provide an early assessment of the severity of COVID-19 epidemics to help stakeholders act swiftly and decisively. Early projections of the transnational spread of SARS-CoV-2 influenced travel restrictions and border closures (Adekunle et al., 2020; McBryde et al., 2020). Model projections based on the infectiousness of SARS-CoV-2 demonstrated its pandemic potential, which guided the global response to and prepared countries for increases in hospitalizations and deaths (Liu et al., 2020; McBryde et al., 2020). Modelling studies that combined historical epidemiological data to project the trend and severity of COVID-19 epidemics for different policy decisions informed stakeholders about the potential effects of interventions before implementation (Koo et al., 2020; Moore et al., 2021; Panovska-Griffiths et al., 2020; [Bibr bib0042][Bibr bib50], Shen et al., 2021a). Models have played a non-negligible role in early outbreak surveillance and policy development. However, none of the previous modelling studies quantified the commencement time and extent of interventions when facing a COVID-19 outbreak of unknown severity and impact on the community. A predictive model that provides timely alerts for intervention commencement and the extent of interventions would have great practical value in curbing COVID-19 outbreaks.

The objective of this study was to establish a predictive model to assist stakeholders in decision-making regarding timely and effective interventions based on limited surveillance data in the early stages of an outbreak. Building on previous models (Bai et al., 2021; Shen et al., 2020a, Shen et al., 2020b, Shen et al., 2021b, Shen et al., 2021c, Shen et al., 2021d; Zhang et al., 2020; Zu et al., 2021), this model integrates existing public health interventions, population vaccination coverage, and the transmissibility of variants. It predicts the severity of the COVID-19 epidemic in the near future and quantifies the critical timing and extent of interventions to allow outbreaks to be contained. Australia was selected as a case study because it has developed a sophisticated COVID-19 surveillance system that reports the number of daily cases with a source of infection (i.e., whether a new diagnosis is linked to a known case) (Australian Government Department of Health, 2021a). This provides essential information to shed light on the extent of viral spread at the community level (Li et al., 2020; Moghadas et al., 2020). The findings of this study could be used to inform decision-making on interventions in Australia and are transferrable to other settings worldwide.

## 2 Materials and methods

### 2.1 Data sources

COVID-19 epidemic data were collected, including the number of daily reported cases (both with known and unknown sources), cumulative confirmed cases, and deaths, based on official reports from the Australian Department of Health (January 25, 2020 to March 12, 2021) (Australian Government Department of Health, 2021a). Satisfactory data from Victoria, New South Wales, the Australian Capital Territory, and Western Australia were collected for analysis, while other states and territories were not included due to a lack of detailed information on the source of confirmed cases (e.g., whether cases were from known clusters). The model was calibrated against the Victorian data (**Supplementary Material** Appendix p11–12 and Figure S5). Data from New South Wales, Australian Capital Territory, and Western Australia were used for model validation (**Supplementary Material** Appendix p17 and Figure S10). Relevant health policies and timelines for COVID-19 interventions were collected from the official website of the Australian Government Department of Health, and social activity data were collected from Google COVID-19 community mobility data (Australian Government Department of Health, 2021a; Google, 2021) (**Supplementary Material** Figures S2–S4).

### 2.2 Model structure and assumptions

A susceptible–infected–recovered compartmental model (**Supplementary Material** Figure S1) was constructed based on published studies (Shen et al., 2020a; Zhang et al., 2020) to simulate the transmission of SARS-CoV-2 in the Australian population (parameters in **Supplementary Material** Table S1). The five public health interventions reported below were integrated into the model (**Supplementary Material** Appendix p2–11).

Face mask use reduces the probability of transmission in each exposure. It was estimated that there would be a reduction of 75% (95% confidence interval 50–95%) in a single exposure with the presence of a face mask (Chu et al., 2020; Howard et al., 2021; MacIntyre et al., 2008).

Social distancing reduces the average number of daily contacts in public spaces. Based on proportional deviations of real-time mobility from the pre-epidemic level in public places from Google COVID-19 community mobility data, we estimated the average number of daily contacts at various levels of social distancing restrictions.

Contact tracing enables a proportion of all close contacts of confirmed cases to be quarantined and tested. The model estimated that contact tracing in Australia would reach 80% of close contacts of the diagnosed individuals. Among the identified close contacts, approximately 20% of respondents would be uncooperative, and 60% of recall information might be biased (Alsubaie et al., 2019; Dyani, 2020).

Ideally, voluntary testing would be performed for individuals who believe that they have been in close contact with infected individuals and may be at risk of infection. This question was simplified by assuming that approximately 0.09–0.2% of the Australian population were receiving voluntary testing daily, according to the reported cumulative number of COVID-19 voluntary tests over the past 7 days and the population size in Australia.

Vaccination will protect the proportion of the population in receipt of the vaccine who develop an immune response. The population vaccination effectiveness was estimated to be about 82.2% by weighing the percentages for the supply of COVID-19 vaccines in Australia (Australian Government Department of Health, 2021b; Baden et al., 2021; Polack et al., 2020; Voysey et al., 2021). A 2% and 10% reduction in the efficacy of the existing vaccines against the Alpha and Delta variants, respectively, was also assumed (Lopez Bernal et al., 2021; Sheikh et al., 2021).

### 2.3 Output indicators and analysis

#### 2.3.1 Undocumented cases during outbreaks

Undocumented cases represent a potential risk of further community transmission of SARS-CoV-2. ‘Undocumented cases’ were defined as asymptomatic infections, pre-symptomatic infections, and symptomatic infections before diagnosis. The association between reported daily locally acquired cases and model-estimated potential undocumented cases in past Australian outbreaks was explored and a significant linear relationship was found between them (**Supplementary Material** Appendix p13–15 and Figures S6–S9).

#### 2.3.2 Basic and effective reproduction numbers

**Figure 1:**
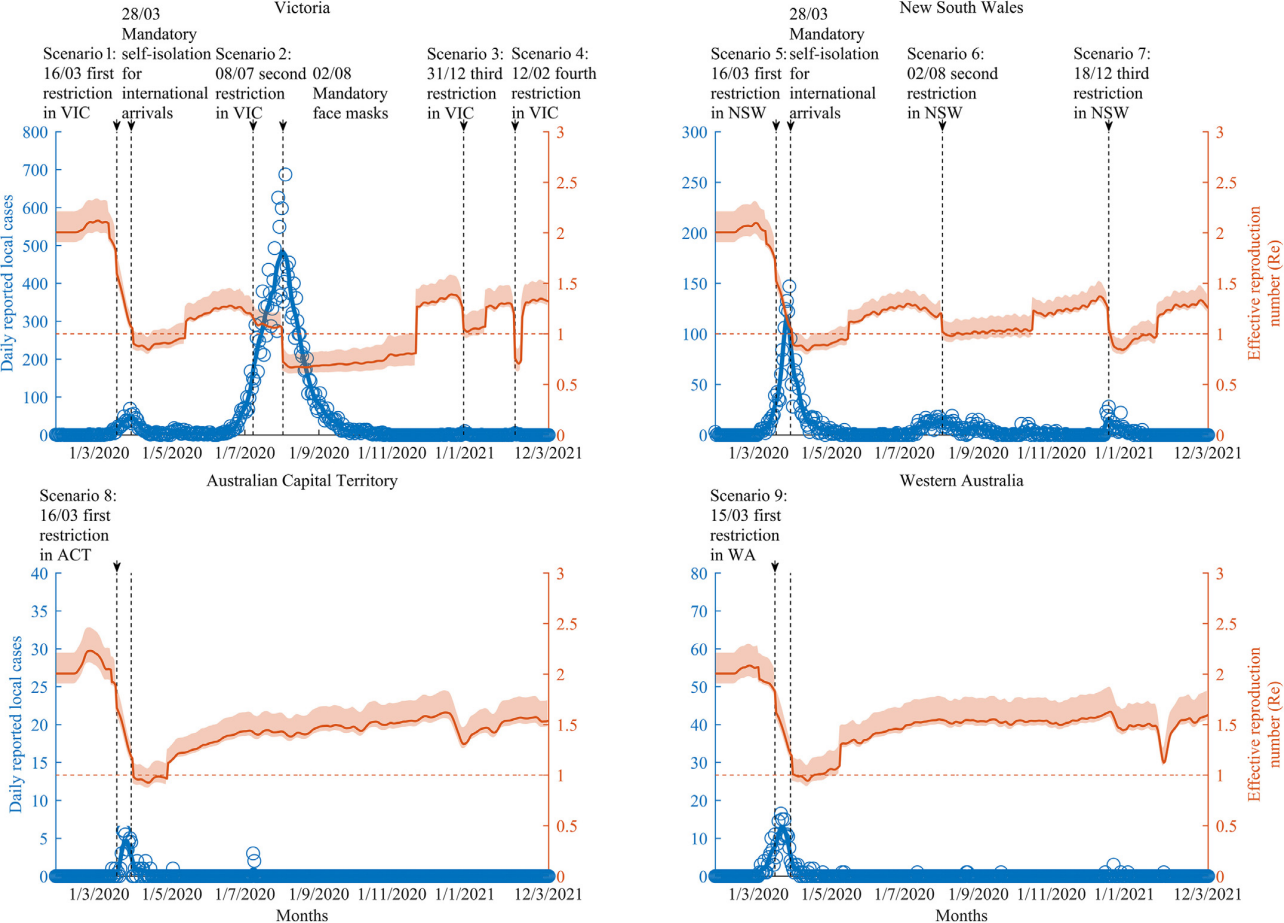
Historical outbreaks of the COVID-19 epidemic and R_e_ in four Australian states (January 25, 2020 to March 12, 2021). From March 16, 2020, all arrivals in Australia were required to be in self-imposed isolation, and this became mandatory from March 28, 2020. Therefore, overseas cases were counted as local cases up to March 16, 2020; 50% of overseas cases were counted as local cases between March 17, 2020 and March 28, 2020; and overseas cases were no longer counted as local cases after March 28, 2020. Abbreviations: VIC, Victoria; NSW, New South Wales; ACT, Australian Capital Territory; WA, Western Australia.

The basic reproduction number (R_0_) represents the average number of secondary cases generated by a typical infectious case when it is introduced into a fully susceptible population (van den Driessche and Watmough, 2002). R_0_ was estimated to be 2.01 (1.91–2.21) in Australia, consistent with previous findings (1.40–2.27) (Price et al., 2020; Rockett et al., 2020; Stapelberg et al., 2021). The effective reproduction number (R_e_) measures the actual transmissibility of an infectious disease in a population with the presence of public health interventions (Bo et al., 2021; Yabe et al., 2020). The time-varying R_e_ of COVID-19 by Australian state is presented in Figure 1 (see **Supplementary Material** Appendix p16 for the detailed derivation and calculation).

#### 2.3.3 Predicted number of reported cases in the next 7 days

The risk of a COVID-19 outbreak was assessed by predicting the number of secondary cases potentially caused by undocumented cases over the next 7 days. First, R_e_ was estimated (Figure 1), which represents the average number of secondary cases caused by a case during an average 14-day infectious period (i.e., the weighted period of the average interval from infection to isolation for symptomatic individuals and the average interval from infection to spontaneous recovery for asymptomatic individuals; **Supplementary Material** Appendix p16). Second, the number of undocumented cases was estimated based on the daily number of unknown-source and known-source cases through the linear relationships (**Supplementary Material** Figure S8). Third, the overall number of secondary cases over the next 14 days was estimated by multiplying R_e_ with the number of undocumented cases. Dividing it by 2 gave the estimate of the number of reported cases over a 7-day period.

#### 2.3.4 Critical timing for intervention commencement

The number of daily reported locally acquired cases was used as an indicator to inform the critical timing for intervention commencement. The turning point (peak) of the epidemic will occur approximately 1 week after commencement of the intervention (Qiu et al., 2021; Triukose et al., 2021). Moreover, the number of daily reported cases will likely continue to rise until R_e_ is reduced below 1. Therefore, during the time gap between intervention commencement and when R_e_ falls below 1, confining the number of daily reported cases to a manageable level (e.g. ≤10 cases/day) may avoid overloading the healthcare capacity and reduce the impacts on economic activities. We defined the number of reported cases that would trigger intervention commencement as the critical timing for intervention implementation, at which time commencing effective interventions (reducing R_e_ to below 1) could maintain the average number of daily reported cases over a 7-day period below 10. cases to be the critical timing for intervention implementation (**Supplementary Material** Appendix p17).

### 2.4 Uncertainty and sensitivity analysis

A probabilistic sensitivity analysis was conducted based on 1000 simulations to accommodate the uncertainty of model parameters and determine the 95% confidence interval of the reproduction number. In addition, multiple scenarios were established to explore the impact of the transmissibility of SARS-CoV-2 variants, vaccination coverage, and effectiveness of face mask use.

## 3 Results

### 3.1 Historical outbreaks of the COVID-19 epidemic and R_e_

**Figure 2:**
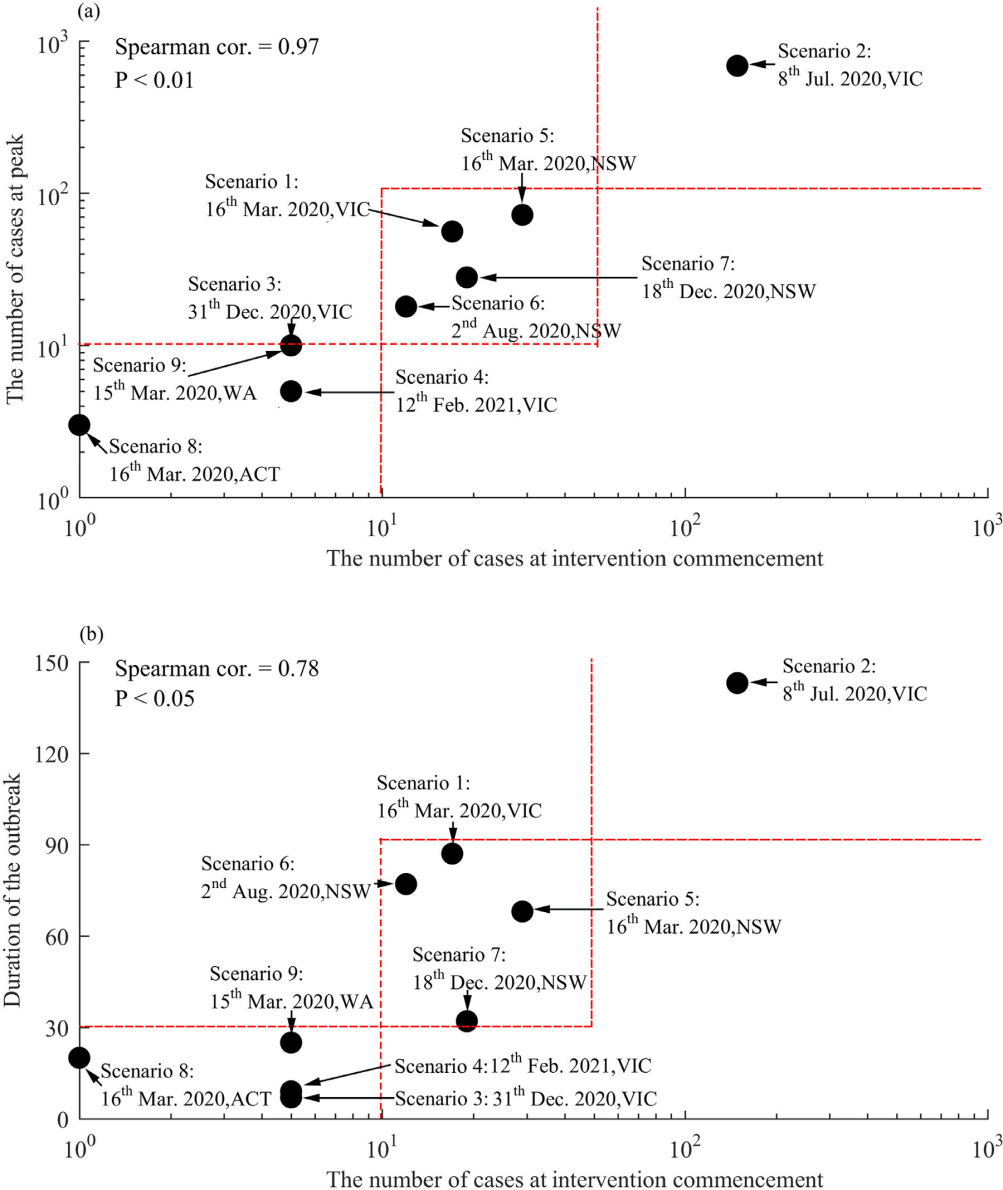
Association between the reported cases on the day interventions commenced and the subsequent peak size and duration of the outbreak. Abbreviations: VIC, Victoria; NSW, New South Wales; ACT, Australian Capital Territory; WA, Western Australia.

Nine outbreaks occurring in the four Australian states were identified from January 25, 2020 to March 12, 2021 (Table 1 and Figures 1 and 2). It was observed that the reported cases on the day interventions commenced were highly correlated with the subsequent peak size and duration of the outbreak (Figure 2). Across the nine outbreaks, there were four occasions on which the state government intervened when the number of daily reported cases was below 10. In these cases, the subsequent peak size of the outbreak was limited (1–10 cases) and the outbreak was contained within a month. In contrast, on four occasions, the state government intervened when the number of daily cases was between 10 and 30. The subsequent outbreak peak was substantial (<100 cases) and the outbreak was contained within 3 months. On one occasion, intervening late at a daily reported case number of 149 resulted in a very high outbreak peak of 687 cases and an outbreak duration of almost 5 months (Table 1 and Figures 1 and 2). Based on these past outbreaks, 10 cases per day was used as a manageable threshold.

**Table 1:** Details of the nine COVID-19 outbreaks in the four Australian states.

COVID-19 outbreak	Restriction period	Interventions implemented	Daily number of reported locally acquired cases at intervention commencement	Estimated undocumented cases at intervention commencement	Number of reported locally acquired cases at subsequent outbreak peak	Duration from the first locally acquired case to three consecutive days without new cases	Estimated R_e_ at intervention commencement(95% CI)	Number of days required to reduce R_e_ to below 1	Minimum R_e_ during the restriction period(95% CI)	Number of days required to reach minimum R_e_
VIC 1 (Scenario 1)	16/03/2020 to 13/05/2020 (58 days)	• State of emergency declaration; • Non-essential business closure; • Mandatory self-isolation for travelers; • Stay-at-home restrictions	17 cases*	199 (65–283) cases*	56 cases	87 days	1.83 (1.80–2.07)	13 days	0.82 (0.78–0.94)	26 days
VIC 2 (Scenario 2)	08/07/2020 to 13/09/2020 (67 days)	• Stay-at-home restrictions; • Daily curfew from 8:00 pm to 5:00 am; • “Five kilometer rule”; • Compulsory wearing of face mask indoors and outdoors	149 cases	2230 (2197–2357) cases	687 cases	143 days	1.17 (1.14–1.32)	24 days	0.63 (0.56–0.76)	32 days
VIC 3 (Scenario 3)	31/12/2020 to 18/01/2021 (18 days)	• Reduction of the limit on the number of people gathering in the home from 30 to 15; • Compulsory wearing of face mask in public indoor spaces	5 cases	50 (49–53) cases	10 cases	7 days	1.28 (1.24–1.44)	3 days	0.98 (0.91–1.14)	4 days
VIC 4 (Scenario 4)	12/02/2021 to 17/02/2021 (5 days)	• Stay-at-home restrictions; • “Five kilometer rule”; • Compulsory wearing of face mask indoors and outdoors	5 cases	27 (26–29) cases	5 cases	9 days	1.19 (1.15–1.33)	1 day	0.65 (0.57–0.81)	4 days
NSW 1 (Scenario 5)	16/03/2020 to 15/05/2020 (60 days)	• Non-essential business closure; • Mandatory self-isolation for travelers; • Stay-at-home restrictions	29 cases*	428 (218–554) cases*	72 cases	68 days	1.74 (1.71–1.96)	13 days	0.81 (0.77–0.93)	26 days
NSW 2 (Scenario 6)	02/08/2020 to 16/10/2020 (75 days)	• Strongly encouraging greater use of masks in public indoor spaces	12 cases	153 (151–162) cases	18 cases	77 days	1.19 (1.15–1.33)	2 days	0.94 (0.88–1.09)	8 days
NSW 3 (Scenario 7)	18/12/2020 to 29/01/2021 (42 days)	• Stay-at-home restrictions; • Compulsory wearing of face mask in public indoor spaces	19 cases	200 (197–211) cases	28 cases	32 days	1.27 (1.23–1.43)	3 days	0.81 (0.77–0.93)	12 days
ACT 1 (Scenario 8)	16/03/2020 to 08/05/2020 (53 days)	• State of emergency declaration; • Non-essential business closure; • Mandatory self-isolation for travelers; • Stay-at-home restrictions	1 case*	5 (3–6) cases*	3 cases	20 days	1.89 (1.86–2.14)	14 days	0.89 (0.84–1.02)	26 days
WA 1 (Scenario 9)	15/03/2020 to 27/04/2020 (43 days)	• State of emergency declaration; • Non-essential business closure; • Mandatory self-isolation for travelers; • Stay-at-home restrictions	5 cases*	77 (18–179) cases*	10 cases	25 days	1.83 (1.80–2.06)	14 days	0.91 (0.85–1.07)	26 days

Abbreviations: VIC, Victoria; NSW, New South Wales; ACT, Australian Capital Territory; WA, Western Australia; CI, confidence interval; R_e_, effective reproduction number.

⁎ From 16 March 2020, all arrivals in Australia were required to be in self-imposed isolation, and this became mandatory from 28 March 2020. Therefore, we counted overseas cases as local cases up to 16 March 2020; 50% of overseas cases as local cases between 17 March 2020 and 28 March 2020; and overseas cases after 28 March 2020 were no longer counted as local cases.

The interval between intervention commencement and a reduction in R_e_ to below 1 was reduced over time (Table 1 and Figure 1). In four recent outbreaks (after the second outbreak in Victoria, the most severe outbreak in Australia), R_e_ was reduced to near or below 1 less than 7 days after intervention commencement.

### 3.2 Surveillance for outbreak severity

**Figure 3:**
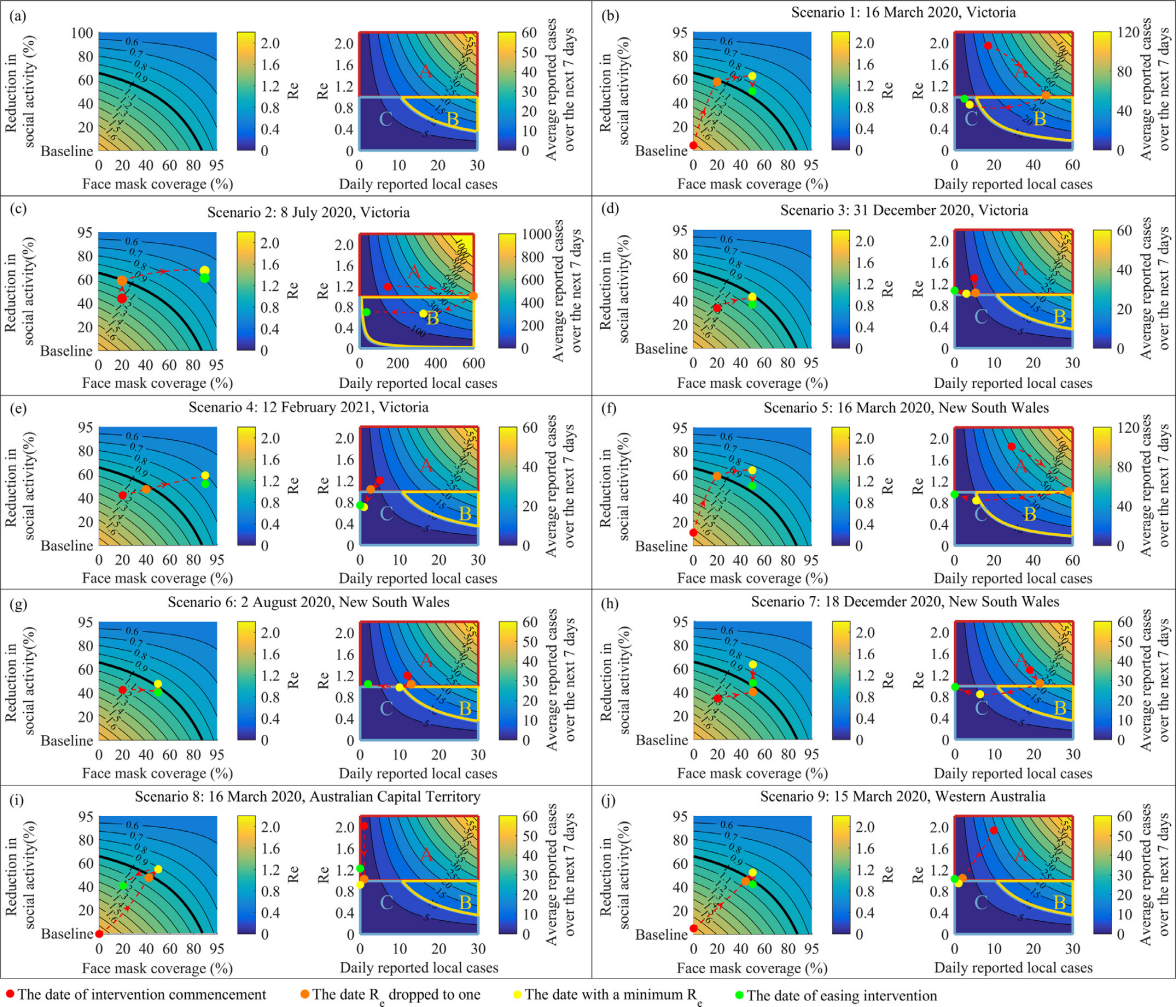
Trajectories of changes of intervention, R_e_, and the predicted outbreak severity for the past COVID-19 outbreaks in four Australian states. From March 16, 2020, all arrivals in Australia were required to be in self-imposed isolation, and this became mandatory from March 28, 2020. Therefore, overseas cases were counted as local cases up to March 16, 2020; 50% of overseas cases were counted as local cases between March 17, 2020 and March 28, 2020; and overseas cases were no longer counted as local cases after March 28, 2020. Abbreviation: R_e_, effective reproduction number.

A surveillance interface was developed to predict the potential outbreak severity based on the current daily reported cases and the extent of public health interventions. The first panel in Figure 3a illustrates how the combinations of various levels of reduction in social activity and face mask coverage might impact R_e_ under the baseline scenario (in a wildtype-dominant epidemic with contact tracing, voluntary testing, but no vaccination). It was observed that reducing social activity by two-thirds of the pre-epidemic level or increasing face mask use to at least 77% would reduce R_e_ to below the threshold curve of 1. The second panel in Figure 3a demonstrates the projected average number of daily cases over the next 7 days based on various combinations of R_e_ and the number of daily reported cases. It shows three distinct regions that reflect various epidemic severities. Region A, where R_e_ is ≥1, indicates an uncontrolled and expanding epidemic in the near future; region B, where R_e_ is <1 but the predicted average daily cases over the next 7 days will still exceed a manageable level (e.g. 10 cases/day), indicates a controlled epidemic with a substantial risk of resurgence; region C, where R_e_ is <1 and the predicted average daily cases over the next 7 days is <10, indicates a controlled epidemic with a reducing risk of resurgence.

Figure 3b–j illustrates the trajectories of intervention change and the predicted outbreak severity for the nine investigated outbreaks, from the date of intervention commencement to the date R_e_ dropped to 1, to the date with a minimum R_e_, and to the date of intervention easing. On five occasions, the trajectory of the projected epidemic severity underwent a shift from region A to region B before moving to region C, suggesting a substantial risk of a large and long-lasting outbreak. In contrast, on the remaining four occasions, the trajectory of the projected epidemic severity shifted directly from region A to region C, suggesting a well-controlled outbreak. These were consistent with the actual outbreak outcomes (Table 1 and Figure 1). The number of cases on the day the intervention commenced largely determined whether the trajectory of epidemic severity would pass through region B.

### 3.3 Critical timing for intervention commencement to avoid major outbreaks

**Figure 4:**
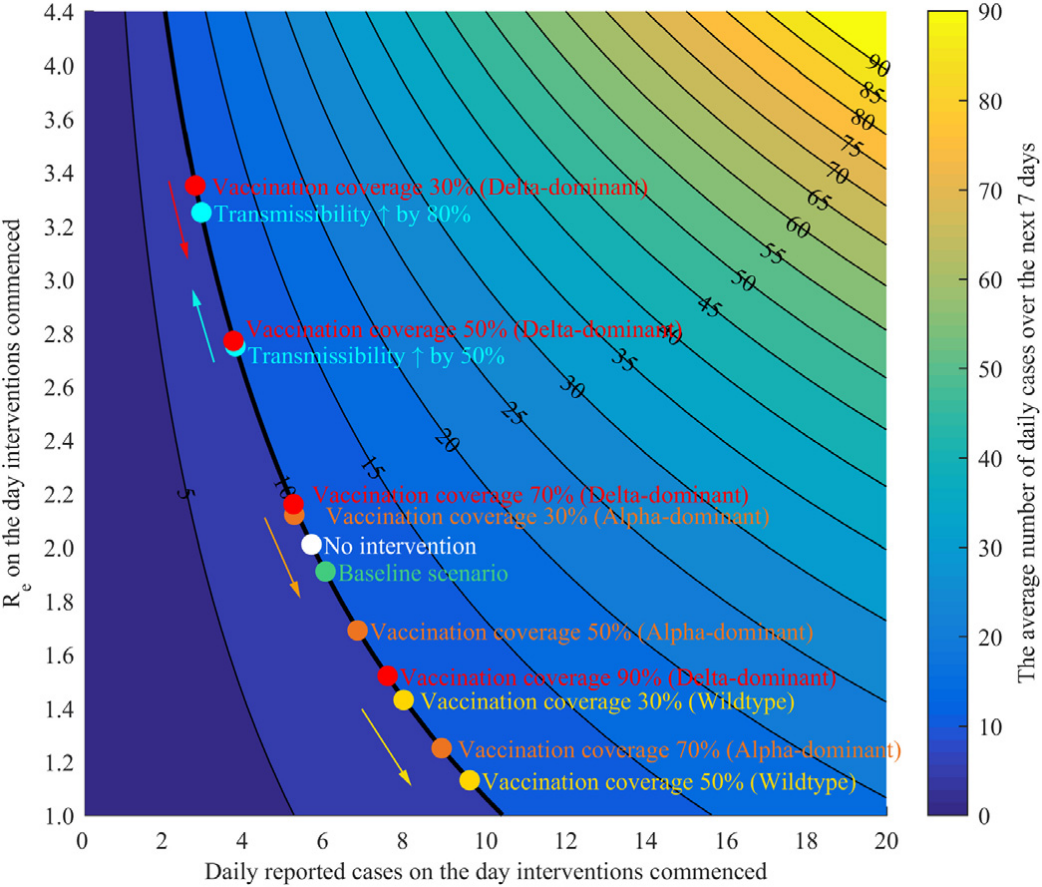
Predicted outbreak severity at various combinations of the number of daily reported cases and R_e_ on the day of intervention commencement. Abbreviation: R_e_, effective reproduction number.

We predicted the average number of daily cases over the first 7 days after interventions at various combinations of the number of daily reported cases and R_e_ on the day of intervention commencement (Figure 4). In the baseline scenario, if the goal was to contain the epidemic to an average of ≤10 daily cases over the next 7 days, the critical number of daily reported cases that should trigger interventions was six.

### 3.4 Critical timing and extent of interventions in epidemics dominated by viral variants

**Figure 5:**
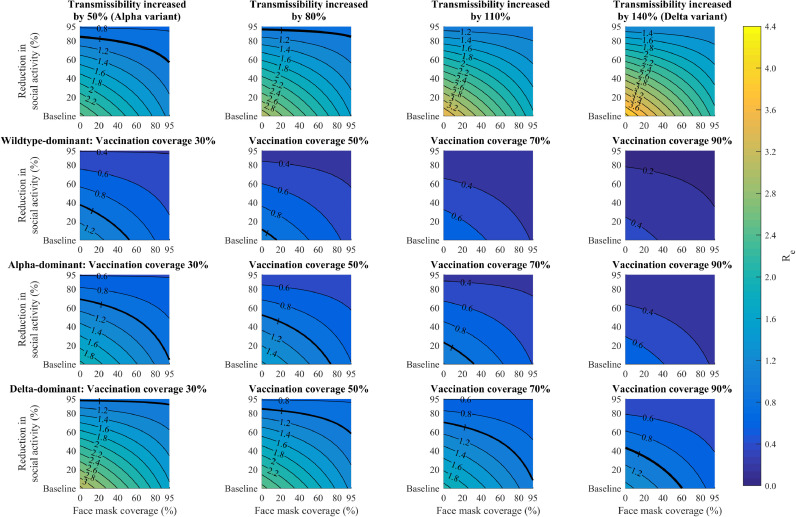
Effect of various levels of reduction in social activity and face mask coverage on R_e_ under different scenarios. Abbreviation: R_e_, effective reproduction number.

If the transmissibility of the novel variants increased by 50%, 80%, 110%, and 140%, a rising or even vanishing threshold curve of R_e_ was observed (R_e_ = 1, Figure 5). This indicates that more stringent combinations of social distancing and face mask use, even in combination with vaccination, would be required to reduce R_e_ below 1. With a 50% (estimated 40–80% for the Alpha variant (Davies et al., 2021; Fort, 2021; Institute of Social and Preventive Medicine (ISPM), University of Bern, 2021)) increase in transmissibility, reducing social activity by 80% of the pre-epidemic level combined with mandatory masks (50% coverage) could reduce R_e_ to 1 without any vaccination. The critical number of cases to trigger intervention commencement would be four to maintain the average number of daily cases over the next 7 days to ≤10 (Figure 4). In contrast, with a 140% (estimated to be 60% (Mahase, 2021) higher for the Delta variant than for the Alpha variant and 140% higher than the wildtype) increase in transmissibility, social distancing and face mask use alone would not be sufficient to reduce R_e_ below 1.

### 3.5 Impact of vaccination coverage on critical timing and extent of interventions

If COVID-19 vaccination coverage reached 30%, 50%, 70%, and 90% in a wildtype-dominant epidemic, a substantial decrease in the threshold curve of R_e_ was observed (R_e_ = 1, Figure 5). The curve disappeared if vaccination coverage exceeded 70%, indicating that social distancing and face mask use restrictions would no longer be necessary if 70% of Australians were vaccinated. However, in an Alpha-dominant epidemic, vaccination coverage would need to reach 90% for social distancing and mask use restrictions to be fully relaxed. In a Delta-dominant epidemic, combinations of vaccination with social distancing and face mask use restrictions would be required to reduce R_e_ to 1. It was found that at 30% vaccination coverage, it would be almost impossible to reduce R_e_ below 1 in combination with existing public health interventions. At 50% vaccination coverage, very strict social distancing (reduced by 70–80% of the pre-pandemic level) combined with face mask use (more than 80%) would still be needed to reduce R_e_ to below 1. To keep R_e_ below 1, 70% vaccination coverage, combined with a 40–50% reduction in social activities and 60–70% face mask use, would be necessary; and at 90% vaccination coverage, it would only require a moderate (40%) reduction in social activities or sustaining 60% face mask use alone.

In all wildtype and variant dominant epidemics, the critical timing for intervention commencement could be delayed with increasing vaccination coverage (Figure 4). In a Delta-dominant epidemic with 70% vaccination coverage, the number of reported cases on the day of intervention commencement could not exceed five cases to maintain the average number of cases over the next 7 days to ≤10. The sensitivity analysis demonstrated that the effectiveness of face masks might affect the vaccination coverage required to reduce R_e_ below 1 in a Delta-dominant epidemic (**Supplementary Material** Figures S11 and S12). Assuming that Australia achieves the target vaccination coverage of 70% (Australian Government, 2021), the ratio of Alpha to Delta variants would also influence the level of social distancing and face mask use required to reduce R_e_ to below 1 in mixed epidemics (**Supplementary Material** Figure S13).

## 4 Discussion

This study identified the critical timing and extent for commencing public health interventions to contain COVID-19 outbreaks in Australia. It was found that in the past Australian outbreaks, the number of reported cases on the day interventions commenced was a strong predictor of the subsequent peak size and duration of the outbreaks. This study demonstrated the critical timing and extent of intervention required to contain the outbreak to a manageable level in different scenarios of variant transmission and vaccination coverage. It was found that in the early phase of an outbreak, containing the prospective epidemic to a low level (≤10 cases/day) would require effective interventions to be commenced before the number of daily reported cases reaches six. Containing an Alpha-dominant epidemic would require more stringent interventions that commence earlier. For the Delta variant, public health interventions alone would not contain the epidemic unless vaccination coverage was ≥70%. In this case, to maintain the prospective epidemic to a low level (≤10 cases/day) would still require effective interventions to be commenced before the number of daily reported cases reaches five.

This study developed a practical model to assist decisions for determining the critical timing and extent of interventions. It appears that this study is the first of its kind to integrate existing public health interventions and epidemic severity to quantify the risk of COVID-19 resurgence. The study also quantified the impact of various potential changes in viral transmissibility and levels of vaccination on the timing and extent of interventions, which is an important consideration given the current epidemic of the Delta variant worldwide. Additionally, the model may be extended to demonstrate future epidemic trends resulting from various combinations of different levels of public health interventions commenced at different time points. Our study will provide stakeholders with intuitive recommendations on the optimal timing of changes in policy and effective combinations of public health interventions, thereby helping to design fit-for-purpose policies.

In this study, it was confirmed that the early commencement of strong public health interventions is critical for containing a COVID-19 outbreak. The findings are echoed in many outbreaks in other settings. For example, in the recent (August 2021) Delta-dominant outbreak in New Zealand, the government declared a Level 4 lockdown and mandatory face mask use interventions immediately after the emergence of five local cases, resulting in rapid containment of the outbreak within a month (Ministry of Health, 2021). In the face of re-emerging community transmission of COVID-19, the results of this study will provide timely alerts for stakeholders on intervention commencement and will be an important component of outbreak surveillance in Australia.

The results are particularly relevant when facing the emergence of more transmissible SARS-CoV-2 variants. The Alpha variant has about 50% (range 40–80%) higher transmissibility than the wildtype (Davies et al., 2021; Fort, 2021; Institute of Social and Preventive Medicine, 2021). It has become dominant in the United States and many parts of Europe, leading to a rebound of the epidemic (Kirby, 2021; New and Emerging Respiratory Virus Threats Advisory Group, 2020). For this variant, our model predicts that stricter measures that include an 80% reduction in social activities and 50% public face mask use would be necessary to contain the epidemic without vaccination, and these restrictions need to be implemented earlier, when there are only four reported daily cases. In contrast, for the Delta variant, with 60% higher transmissibility than the Alpha variant (Mahase, 2021), combinations of vaccination and other interventions would be necessary to contain it (Figure 5). However, this study encouragingly illustrates that expanding COVID-19 vaccination is an effective means of COVID-19 control and socioeconomic recovery. Nevertheless, concerted efforts with other public health interventions are still necessary if high vaccination coverage cannot be guaranteed in the short term or when facing more transmissible new variants. Our study will continue to provide important information for timely changes in public health interventions to help stakeholders make the most appropriate decisions as more transmissible variants emerge and vaccination coverage continues to increase.

This study has several limitations. First, historical epidemics were simulated based on four Australian states but excluding Queensland, which experienced a significant outbreak in March–April 2020. This is because the state's official reports did not include information on whether a diagnosed case was from a known or unknown source and hence could not inform our model. Second, environmental differences were not considered, which are likely to play a role and differ between states. Third, regarding the completion date of this study, reliable data on the transmissibility and mortality of the novel variants of SARS-CoV-2 and the effectiveness of the COVID-19 vaccine against new variants are still under investigation. This study was conducted with limited availability of these data. Finally, we did not differentiate between the nature of the new cases. Cases in the same household as a known case and those who were isolated at the time of their diagnosis will be different from cases that are not linked to known cases and who were not isolated at the time of their diagnosis. Further individual-based modelling studies are necessary to explore the impact of new cases of different nature on the severity of the outbreak.

This study quantified, for the first time, the critical timing and extent of public health interventions that would effectively control an outbreak. It provides stakeholders with intuitive recommendations for taking early and decisive action and, therefore, has important implications for facilitating the achievement of the ambitious goal of rapid and complete control of the COVID-19 outbreak.

## Author contributions

LZ and ZZ designed the study. ZZ designed and constructed the model. LZ, MS, and NS contributed to provide technical and modelling advice throughout the project. ZZ performed the modelled analyses, graphed and interpreted the results. ZZ, XX, ZL, and RL contributed to the collection of data and model parameters. ZZ drafted the manuscript. LZ, CKF, and GZ critically revised the manuscript. All authors reviewed the manuscript and approved the final version.

## Declarations

*Funding source:* LZ is supported by the National Natural Science Foundation of China (grant number 8191101420), Thousand Talents Plan Professorship for Young Scholars (grant number 3111500001), Xi'an Jiaotong University Basic Research and Profession Grant (grant number xtr022019003), and Xi'an Jiaotong University Young Talent Support Program (grant number YX6J004). MS is supported by the National Natural Science Foundation of China (grant number 12171387, 11801435), China Postdoctoral Science Foundation (grant number 2018M631134, 2020T130095ZX), the Fundamental Research Funds for the Central Universities (grant number xjh012019055), Natural Science Basic Research Program of Shaanxi Province (grant number 2019JQ-187), and Young Talent Support Program of Shaanxi University Association for Science and Technology (grant number 20210307). The study was supported by the Bill and Melinda Gates Foundation.

*Ethical approval:* No human subjects were involved in this work and therefore ethical approval was not required for the development of this manuscript.

*Conflict of interest:* The authors declare no conflict of interest.

## Appendix Supplementary materials

Image, application 1
