# Supplementary material for: Critical timing and extent of public health interventions to control outbreaks dominated by SARS-CoV-2 variants in Australia: a mathematical modelling study
Source: Int J Infect Dis. 2022 Feb;115:154–65. doi: 10.1016/j.ijid.2021.11.024 (PMC8598259; doi:10.1016/j.ijid.2021.11.024)
Supplement: Supplementary file 1 [file mmc1.docx]

**Supplementary Materials**

**Critical timing and extent of public health interventions to control outbreaks dominated by SARS-CoV-2 variants in Australia: a mathematical modelling study**

Zhuoru Zou^a,1^, Christopher K Fairley^a–c^, Mingwang Shen^a^, Nick Scott^d^, Xianglong Xu^a–c^, Zengbin Li^a^, Rui Li^a^, Guihua Zhuang^a,*^, Lei Zhang^a–c,*^

^a^ China-Australia Joint Research Centre for Infectious Diseases, School of Public Health, Xi'an Jiaotong University Health Science Centre, Xi'an, 710061, China

^b^ Melbourne Sexual Health Centre, Alfred Health, Melbourne, VIC 3053, Australia

^c^ Central Clinical School, Faculty of Medicine, Monash University, Melbourne, VIC 3800, Australia

^d^ Burnet Institute, Melbourne, VIC 3004, Australia

^*^Equal contribute to supervision

**Corresponding authors:**

Prof. Lei Zhang. PhD. China-Australia Joint Research Centre for Infectious Diseases, School of Public Health, Xi’an Jiaotong University Health Science Centre, Xi’an, Shaanxi, 710061, China, E-mail: lei.zhang1@monash.edu

Prof. Guihua Zhuang. PhD. China-Australia Joint Research Centre for Infectious Diseases, School of Public Health, Xi’an Jiaotong University Health Science Centre, Xi’an, Shaanxi, 710061, China, E-mail: [zhuanggh@mail.xjtu.edu.cn](mailto:zhuanggh@mail.xjtu.edu.cn)

1. **Model equations and detailed description**
   1. **Model Structure**

We constructed a Susceptible-Infected-Recovered compartmental model based on published studies (Zhang et al., 2020; Shen et al., 2020) to describe the spread of SARS-CoV-2 and the impact of public health interventions. The population is divided into ten compartments (Figure S1a): susceptible individuals (S), asymptomatic infections (A), pre-symptomatic infections (E), symptomatic infections before diagnosis (I), diagnosed individuals with isolation and treatment (T), uninfected individuals among the quarantined close contacts ($Q_{S})$, infected individuals among the quarantined close contacts ($Q_{EA})$, vaccinated individuals (V), recovered individuals (R), and dead individuals (D). The total population size is denoted by N, where $N=S+A+E+I+T+Q_{S}+Q_{EA}+V+R$.


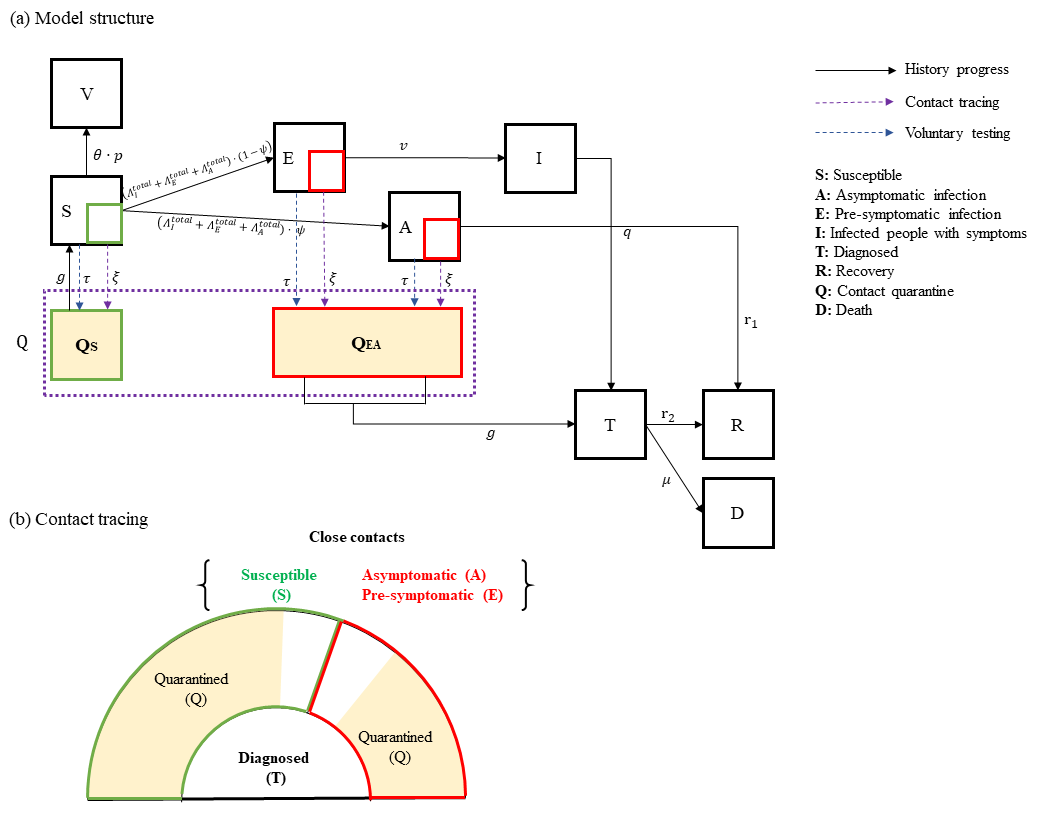


**Figure S1. Schematic diagrams of the transmission of SARS-CoV-2 and contact tracing.**

- 1. **Model equations**

The model is described by the following system of ordinary differential equations. The symbols are defined in the legend following the equations.

| $\dot{S}=-\Lambda_{I}^{total}-\Lambda_{E}^{total}-\Lambda_{A}^{total}-{Cont}_{non-infection}\cdot\xi-\tau\cdot S-\theta\cdot p\cdot S+g\cdot Qs$ | (1) |
| --- | --- |
| $\dot{A}=\left( \Lambda_{I}^{total}+\Lambda_{E}^{total}+\Lambda_{A}^{total} \right)\cdot\psi-r_{1}\cdot A-{Cont}_{infection}\cdot\psi\cdot\xi-\tau\cdot A$ |  |
| $\dot{E}=\left( \Lambda_{I}^{total}+\Lambda_{E}^{total}+\Lambda_{A}^{total} \right)\cdot\left( 1-\psi\right)-v\cdot E-{Cont}_{infection}\cdot\left( 1-\psi\right)\cdot\xi-\tau\cdot E$ |  |
| $\dot{I}=v\cdot E-q\cdot I$ |  |
| $\dot{Q_{S}}={Cont}_{non-infection}\cdot\xi+\tau\cdot S-g\cdot Q_{S}$ |  |
| $\dot{Q_{EA}}={Cont}_{infection}\cdot\xi+\tau\cdot\left( E+A \right)-g\cdot Q_{EA}$ |  |
| $\dot{T}=q\cdot I+g\cdot Q_{EA}-r_{2}\cdot T-\mu\cdot T$ |  |
| $\dot{R}=r_{1}\cdot A+r_{2}\cdot T$ |  |
| $\dot{D}=\mu\cdot T$ |  |

Parameters in the differential equations:

| Compartments symbols | Description |
| --- | --- |
| $S$ | Susceptible individuals |
| $A$ | Asymptomatic infected individuals (cases who never developed any noticeable symptoms during the entire period of their disease) |
| $E$ | Pre-symptomatic infected individuals (cases who have mild symptoms before the onset of symptoms) |
| $I$ | Symptomatic but undiagnosed individuals |
| $Q_{S}$ | Uninfected individuals among the quarantined close contacts |
| $Q_{EA}$ | Infected individuals among the quarantined close contacts |
| $T$ | Diagnosed individuals with isolation and treatment |
| $R$ | Recovered individuals |
| $D$ | Individuals who died from COVID-19-related complications |
| Parameter symbols | Description |
| $\Lambda_{I}^{total}$ | Probability of being infected by exposure to symptomatic infected individuals (I) in public places and households |
| $\Lambda_{E}^{total}$ | Probability of being infected by exposure to pre-symptomatic infected individuals (E) in public places and households |
| $\Lambda_{A}^{total}$ | Probability of being infected by exposure to asymptomatic infected individuals (A) in public places and households |
| $\psi$ | The proportion of asymptomatic infections among newly infected individuals |
| $1/v$ | The mean incubation time (days) |
| $1/q$ | The interval from symptom onset to isolation in hospital or quarantine (days) |
| $1/g$ | The interval from testing to diagnosis (days) |
| $1/r_{1}$ | The mean time from infection to recovery for asymptomatic infected individuals (days) |
| $1/r_{2}$ | The mean time from diagnosis to recovery for symptomatic infected individuals (days) |
| $\mu$ | Disease-induced death rate |
| ${Cont}_{non-infection}$ | Uninfected close contacts of the daily new confirmed cases |
| ${Cont}_{infection}$ | Infected close contacts of the daily new confirmed cases |
| $\xi$ | Effectiveness of contact tracing |
| $\tau$ | The coverage rate of voluntary testing |
| $\theta$ | Efficacy of the COVID-19 vaccine |
| $p$ | The coverage rate of COVID-19 vaccination |

- 1. **Modelling infection progression**

Susceptible individuals could be infected through contact with an infectious source in public places and households. Newly infected individuals would enter the asymptomatic infection compartment (A) and the pre-symptomatic infection compartment (E) according to the proportions $\psi$ (0≤$\psi$≤1) and (1-$\psi)$, respectively. The model assumed that asymptomatic infected individuals would never develop any noticeable symptoms and would not be isolated but later spontaneously recovered at the rate $r_{1}$unless they were diagnosed due to voluntary testing or contact tracing. Pre-symptomatic infected individuals in the incubation period would progress to the symptomatic infection compartment (I) at the rate $v$. Symptomatic infected individuals were assumed to be diagnosed and isolated at the rate $q$ and enter the treatment compartment (T). We also assumed strict isolation so that isolated individuals could not further infect others. Diagnosed individuals would recover at the rate $r_{2}$ or die of COVID-19-related complications at the rate 𝜇.

- 1. **Force of infection, social distancing, and face mask use**

The force of infection was dependent on the infectivity of the virus, the presence and severity of symptoms of the infectious source, the venue of exposure, and public health interventions in the population.

Susceptible individuals may be infected through contact with undocumented cases (sources of infection), including asymptomatic infected individuals (A), pre-symptomatic infected individuals (E), and symptomatic but undiagnosed individuals (I). Force of infection ($\Lambda^{total}$) is given by the sum of probabilities of being infected by exposure to undocumented cases. That is,

|  | $\Lambda^{total}= \Lambda_{A}^{total}+\Lambda_{E}^{total}+\Lambda_{I}^{total}$ | (2) |
| --- | --- | --- |

Transmission may occur in public places and households. Thus, the probability of being infected by exposure to asymptomatic infected individuals ($\Lambda_{A}^{total}$), for example, is the sum of probabilities from these two routes. It can be expressed as:

|  | $\Lambda_{A}^{total}=\Lambda_{A}^{fam}+\Lambda_{A}^{pub}=\beta_{EA}^{fam}\cdot\frac{A}{N_{f}}\cdot S+\beta_{EA}^{pub}\cdot c_{p}(t)\cdot\frac{A}{N}\cdot S$ | (3) |
| --- | --- | --- |
|  | $\Lambda_{E}^{total}=\Lambda_{E}^{fam}+\Lambda_{E}^{pub}=\beta_{EA}^{fam}\cdot\frac{E}{N_{f}}\cdot S+\beta_{EA}^{pub}\cdot c_{p}(t)\cdot\frac{E}{N}\cdot S$ |  |
|  | $\Lambda_{I}^{total}=\Lambda_{I}^{fam}+\Lambda_{I}^{pub}=\beta_{I}^{fam}\cdot\frac{I}{N_{f}}\cdot S+\beta_{I}^{pub}\cdot c_{p}(t)\cdot\frac{I}{N}\cdot S$ |  |

Where,

|  | $\beta_{I}^{fam}=\beta,$ | $\beta_{I}^{pub}=\beta\cdot(1-\rho)\cdot\left( 1-\sigma\cdot m(t) \right)$ | (4) |
| --- | --- | --- | --- |
|  | $\beta_{EA}^{fam}=\beta_{I}^{fam}\cdot\left( 1-\varepsilon\right),$ | $\beta_{EA}^{pub}=\beta_{I}^{pub}\cdot\left( 1-\varepsilon\right)$ |  |

Using the example of being infected by asymptomatic infected individuals ($\Lambda_{A}^{total}$), for household exposure, the probability of being infected ($\Lambda_{A}^{fam}$) is equal to the risk of coming from a household with an asymptomatic infected individual ($\frac{A}{N_{f}}$) multiplied by the average daily probability of being infected in the household ($\beta_{A}^{fam}$); for public places exposure, the probability of being infected ($\Lambda_{A}^{pub}$) is equal to the probability of being exposed to a person who is an asymptomatic infected individual ($\frac{A}{N}$) multiplied by the average daily probability of being infected by contact with an asymptomatic infected individual in public places ($\beta_{A}^{pub}$), and multiplied by the average number of contacts in public places per day ($c_{p}(t)$). Social distancing restrictions will affect $c_{p}(t)$ and thus prevent infection.

Here$N_{f}$ denotes the total number of households, which is equal to the total population size (N) divided by the average household size ($c_{f}$) in Australia. We assumed that undocumented cases are dispersed among different households. Because the prevalence of COVID-19 in Australia is low and the probability of two or more household members being infected simultaneously in various public venues is small. We abbreviated the average daily probability of being infected by contact with the symptomatic infected individual in the household ($\beta_{I}^{fam}$) as $\beta$. Usually, the average frequency of daily person-to-person contacts in public places is less than that within the home. We, therefore, assumed that the average daily probability of being infected by contact with a symptomatic infected individual in a public place ($\beta_{I}^{pub}$) is less than that of being infected by contact with a symptomatic infected individual at home ($\beta_{I}^{fam}$), and donated the percentage reduction as $\rho$ (0≤$\rho$≤1). The parameters $\sigma$ and $m(t)$ denote the effectiveness and coverage rate of face mask use in public places, respectively, which reflect the effect of face mask use on infection prevention. We assumed that for contacts with asymptomatic/pre-symptomatic infected individuals, the average probability of being infected is lower, i.e.$\left( 1-\varepsilon\right)\cdot\beta$ where 0≤$\varepsilon$≤1 denotes the reduction in daily transmission probability.

- 1. **Contact tracing**

Contact tracing enabled a proportion of all close contacts of confirmed cases to be quarantined and tested (Figure S1b). We simulated the effect of contact tracing in three steps.

First, the number of close contacts for newly diagnosed cases in public places and households was calculated according to the definition of close contacts (Integrated Disease Surveillance Programme National Centre for Disease Control, 2020; Centers for Disease Control and Prevention, 2021) using the following formula:

|  | ${Cont}_{I}^{fam}=I\cdot q\cdot\left( c_{f}-1 \right)$ | (5) |
| --- | --- | --- |
|  | ${Cont}_{I}^{pub}=I\cdot q\cdot c_{p}(t)\cdot t_{1}$ |  |
|  | ${Cont}_{EA}^{fam}=Q_{EA}\cdot g\cdot\left( c_{f}-1 \right)$ |  |
|  | ${Cont}_{EA}^{pub}=Q_{EA}\cdot g\cdot c_{p}(t)\cdot t_{2}$ |  |

Where ${Cont}_{I}^{fam}$ and ${Cont}_{I}^{pub}$ indicate the total number of close contacts in households and public places for new cases diagnosed due to symptoms. Similarly, ${Cont}_{EA}^{fam}$ and ${Cont}_{EA}^{pub}$ indicate the total number of close contacts in households and public places for new asymptomatic/pre-symptomatic cases diagnosed due to testing and quarantine. The total number of close contacts was estimated by multiplying the number of new diagnoses by the average number of close contacts per individual. $I\cdot q$ and $Q_{EA}\cdot g$ denote the number of daily new cases diagnosed from symptomatic infected individuals and asymptomatic/pre-symptomatic infected individuals, respectively (Figure S1a). For each confirmed case, the number of close contacts from the household is the number of family members other than the case, i.e., $c_{f}-1$; the number of close contacts from public places is the product of the average daily number of close contacts in public places$(c_{p}(t))$ and the number of tracing days. The parameters $t_{1}$ and $t_{2}$ represent the number of tracing days for symptomatic cases and asymptomatic/pre-symptomatic cases. According to the CDC,^3,4^ “an infected person can spread SARS-CoV-2 starting from 2 days before to 14 days after the onset of symptoms (or, for asymptomatic patients, 2 days before to 14 days after the date sample collection), if not isolated earlier”. The model assumed that symptomatic infected individuals will seek medical attention and be isolated after the onset of symptoms, and that asymptomatic infected individuals will be isolated when they are tested as close contacts. Thus, $t_{1}$ is equal to the interval from symptom onset to isolation in the hospital ($1/q$) plus two days; $t_{2}$ is equal to two days.

Second, we estimated the number of infected individuals among close contacts (${Cont}_{infection}$) and uninfected individuals among close contacts (${Cont}_{non-infection}$) according to the force of infection, expressed as:

|  | ${Cont}_{infection}=$  $\beta_{I}^{fam}\cdot{Cont}_{I}^{fam}+\beta_{I}^{pub}\cdot\frac{1}{t_{1}}\cdot{Cont}_{I}^{pub}+\beta_{EA}^{fam}\cdot{Cont}_{EA}^{fam}+\beta_{EA}^{pub}\cdot\frac{1}{t_{2}}\cdot{Cont}_{EA}^{pub}$ | (6) |
| --- | --- | --- |
|  | ${Cont}_{non-infection}=$  $\left( 1-\beta_{I}^{fam} \right)\cdot{Cont}_{I}^{fam}+\left( 1-\beta_{I}^{pub} \right)\cdot\frac{1}{t_{1}}\cdot{Cont}_{I}^{pub}+\left( 1-\beta_{EA}^{fam} \right)\cdot{Cont}_{EA}^{fam}+$  $(1-\beta_{EA}^{pub})\cdot\frac{1}{t_{2}}\cdot{Cont}_{EA}^{pub}$ |  |

($\beta_{I}^{fam}\cdot{Cont}_{I}^{fam}+\beta_{EA}^{fam}\cdot{Cont}_{EA}^{fam}$) denote the average daily number of infected close contacts from households and ($\beta_{I}^{pub}\cdot\frac{1}{t_{1}}\cdot{Cont}_{I}^{pub}+\beta_{EA}^{pub}\cdot\frac{1}{t_{2}}\cdot{Cont}_{EA}^{pub}$) denote the average daily number of infected close contacts from public places.

Third, depending on the effectiveness of contact tracing (i.e., the ability to detect and quarantine all close contacts, denoted as $\xi$), a proportion of infected close contacts (${Cont}_{infection}\cdot\xi$) would be diagnosed and isolated, while a proportion of uninfected close contacts (${Cont}_{non-infection}\cdot\xi$) would be tested and quarantined but later returned to the susceptible compartment.

- 1. **Voluntary testing and vaccination**

We assumed that a proportion of individuals would be voluntarily tested according to the coverage rate (denoted as $\tau$). Susceptible individuals who have undergone voluntary testing would return to the susceptible compartment (S) after the testing to diagnosis interval. In contrast, asymptomatic/pre-symptomatic infected individuals who have undergone voluntary testing would be diagnosed after the testing to diagnosis interval and thus strictly isolated.

Individuals who have been vaccinated and have developed immune protection would enter the vaccination compartment (V). We assumed that this population would not be able to be infected in the short term.

1. **Data and parameter estimation**
   1. **Epidemiological data**

We searched historical outbreak data for COVID-19 from the official website of the Australian Department of Health for the period 25 January 2020 to 12 March 2021, including the number of daily reported cases (both with known and unknown sources), cumulative confirmed cases, and deaths. Because some states and territories did not report information on the source of confirmed cases (e.g., whether cases were from known clusters), satisfactory data from Victoria, New South Wales, the Australian Capital Territory, and Western Australia were collected for analysis. We calibrated the model with relevant data from Victoria. Further, we verified the reliability of the model outputs with relevant data from New South Wales, the Australian Capital Territory, and Western Australia, respectively.

The epidemiological data from the four states mentioned above were presented in Figure S2. Confirmed cases are classified according to their source as overseas cases, locally known cases and locally unknown cases. From 16 March 2020, all arrivals in Australia were required to be in self-imposed isolation for 14 days, which became mandatory from 28 March 2020. Therefore, we considered that the activity of overseas cases was unrestricted until 16 March 2020, and overseas cases had the same potential for community transmission as locally acquired cases of known sources. We assumed that 50% of overseas cases might comply with strict isolation between 17 March 2020 and 28 March 2020. We also assumed that all overseas cases might be in strict isolation after 29 March 2020, so they contributed only to the number of cases but not community transmission.


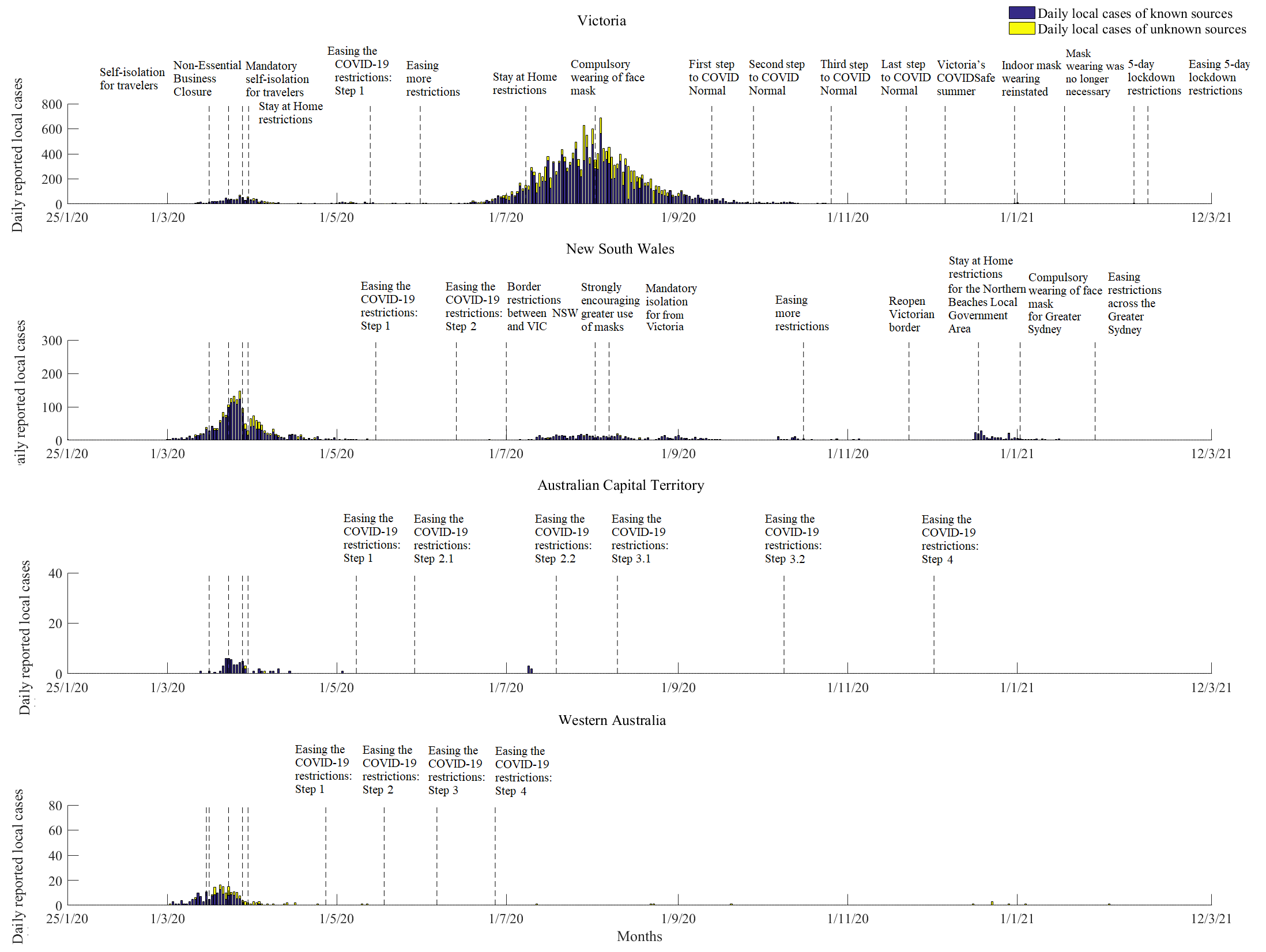


**Figure S2. COVID-19 epidemiological data and public health interventions** **in four Australian states (25 January 2020 – 12 March 2021).**

Figure S3 illustrated the proportion of daily unknown-source cases to total daily locally acquired cases in the historical outbreaks in the four states. We found that about 20% of locally acquired cases in Australia were of unknown sources. This proportion had large fluctuations, especially when the number of daily cases was low.


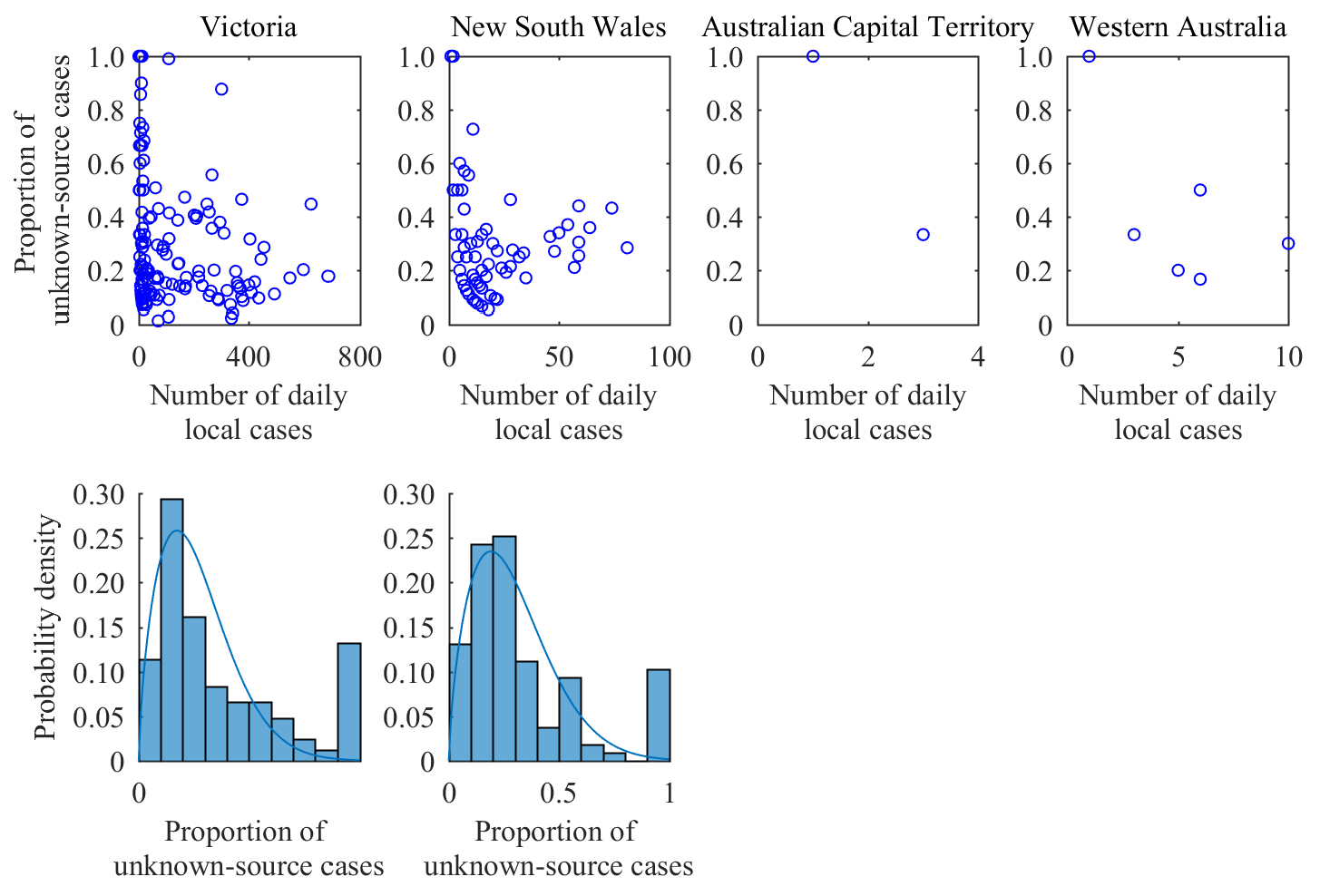


**Figure S3. The proportion of unknown-source cases to total locally acquired cases in historical outbreaks in four Australian states.**

- 1. **Data related to public health interventions**

1. **Reduction in social activity and average number of daily close contacts**

The core elements and timelines of relevant public health policies implemented by each Australian state to control the spread of SARS-CoV-2 were collected and presented in Figure S2. To assess the impact of social distancing restrictions on social activities, we analysed Google COVID-19 community mobility data and obtained changes in mobility in public places (Google, 2021). We expressed mobility changes as proportional deviations from levels for the baseline (Figure S4). It can be seen that policies influenced mobility in public places and that mobility decreased as restrictions were imposed and increased as restrictions were relaxed.


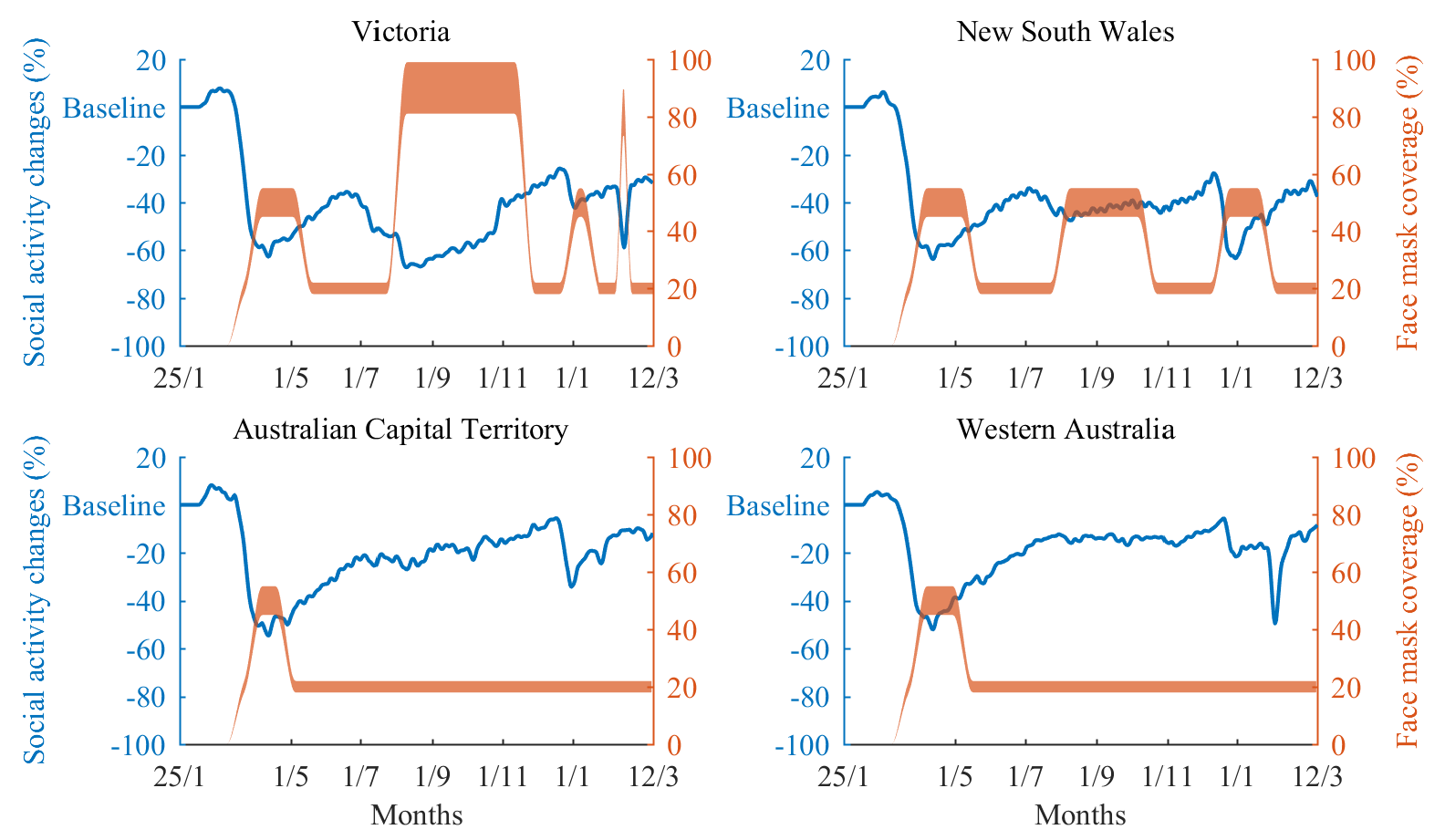


**Figure S4 Social activity changes and face mask coverage rates in four Australian states (25 January 2020 – 12 March 2021).**

We estimated that the average daily number of close contacts for individuals in public places in Australia without social distancing restrictions was 19, based on previous reports (Fitzhugh Mullan Institute for Health Workforce Equity, 2020). Further, we estimated the real-time average daily number of close contacts in public places based on the mobility changes in public places to simulate the impact of social distancing restrictions. According to the Australian Bureau of Statistics, the average household size is three. Therefore, the average number of close contacts in a household was estimated to be two.

1. **Effectiveness and coverage rate of face mask use**

The effectiveness of face mask use in preventing infection was estimated to be 75% (95% CI: 50–95%), based on relevant meta-analysis against COVID-19 (Chu et al., 2020; Howard et al., 2021; MacIntyre et al., 2008). In the context of the COVID-19 pandemic, the rate of face mask use in public places in Australia is around 10–30%, according to the report from the global health research centre at the University of Washington (Institute for Health Metrics and Evaluation, 2021). We assumed that face mask use in public places would increase spontaneously to about 50% when the lockdown was implemented. During mandatory face mask measures in Victoria, the face mask coverage in public places was estimated to reach 80–100%. The ranges of face mask coverage rates over time were estimated based on the relevant public health policies and were displayed in Figure S4.

1. **Effectiveness of contact tracing, rate of voluntary testing, and vaccine efficacy**

The effectiveness of contact tracing may depend on various factors, such as willingness to cooperate, recall bias, availability of contact tracers, and quarantine compliance. Our model estimated that approximately 25% (95% CI: 23.7–26.8%) of the theoretical total close contacts were tested and strictly quarantined in Australia by model calibration. This means that contact tracing in Australia would reach 80% of close contacts of the diagnosed individuals. Among the identified close contacts, approximately 20% of respondents were uncooperative, and 60% of recall information might be biased (Dyani, 2020; Alsubaie et al., 2019).

Based on the cumulative number of COVID-19 voluntary tests over the past 7 days and the population size reported by the Australian Government Department of Health, we estimated that 0.09%–0.2% of the Australian population would receive voluntary testing each day.

According to Australia’s vaccine agreements, the Pfizer/BioNTech vaccine (40 million doses available), the Oxford/AstraZeneca vaccine (53.8 million doses available), and the Moderna vaccine (25 million doses available) will account for 33.7%, 45.3%, and 21.0% of COVID-19 vaccination in Australia, respectively (Australian Government Department of Health, 2021). The efficacy of the Pfizer/BioNTech vaccine, the Oxford/AstraZeneca vaccine, and the Moderna vaccine has been reported to be 95% (90.3–97.6%), 67.1% (52.3–77.3%), and 94.1% (89.3–96.8%), respectively (Baden et al., 2021; Polack et al., 2020; Voysey et al., 2021). We hence estimated the weighted population vaccination effectiveness to be about 82.2%. Recent studies have shown that the existing vaccines remain equally effective in preventing clinical severities in patients infected with Alpha and Delta variants. Still, there was a slight decrease in effectiveness against infection. The efficacy of the Pfizer/BioNTech vaccine reduced to 93.4% (90.4–95.5%) for Alpha and 87.9% (78.2–93.2%) for Delta variant (Lopez Bernal et al., 2021; Sheikh et al., 2021; Stowe et al., 2021). The efficacy of the Oxford/AstraZeneca vaccine reduced to 66.1% (54.0–75.0%) for Alpha and 59.8% (28.9–77.3%) for Delta variant (Lopez Bernal et al., 2021; Sheikh et al., 2021; Stowe et al., 2021). We, therefore, assumed a 2% and 10% reduction in the efficacy of the vaccine against Alpha and Delta.

- 1. **Model calibration and transition probability between model compartments**

Transition probabilities in the model were derived mainly from the published literature. Model calibration was carried out to determine a set of uncertain parameters associated with force of infection. The parameters were summarized in Table S1. We first obtained plausible initial ranges for parameters through an extensive literature review and then obtained the good-fitting parameter sets by model calibration. We calibrated the model by comparing the model-simulated epidemic indicators with the observed data (calibration targets), including the number of daily reported cases, the number of daily known-source cases, the number of daily unknown-source cases, and cumulative deaths, from historical outbreaks in Victoria.

In this process, we employed a ‘genetic algorithm’ (McCall, 2005) to search within the model parameters’ initial ranges to determine the ‘good-fitting’ parameter sets based on the goodness-of-fit score and the likelihood ratio test. We performed about 1,000,000 simulations in total. In each simulation, one value for each parameter was randomly extracted from its initial range, and a set of input values was formed. With this set of input values, the compartmental model was run to produce the outputs compared with the calibration targets. A goodness-of-fit score was calculated by summing the log-likelihoods. Goodness-of-fit scores were assumed to follow a chi-square distribution with the number of degrees of freedom equal to the number of calibration targets. Based on this distribution, ‘good-fitting sets’ of model parameters were identified using the likelihood ratio test, comprising those sets that did not produce a non-inferior fit compared with the best-fitting set (using an alpha level of 5%) (Kim et al., 2007). We ranked the goodness-of-fit scores of the ‘good-fitting sets’ in ascending order and retained the top 1000 sets of best goodness-of-fit. The best-fitting set was the set with the lowest goodness-of-fit score whose simulated outputs were closest to the calibration targets (Table S1). The best-fitting set was introduced into the model as the base-case values of parameters, and the 1000 ‘good-fitting sets’ were used for sensitivity analyses. All analyses and simulations were performed in MATLAB R 2019a.

**Table S1. Transition probabilities between model compartments**

| Parameter | Initial range and reference | Best-fitting set | Range |
| --- | --- | --- | --- |
| The average probability of being infected per day by contact with a symptomatic infected individual in the household ($\beta$) | 0.01–0.05 (Shen et al., 2020) | 0.0285 | 0.027–0.032 |
| Percentage reduction in the average daily probability of being infected by contact with an infectious individual in a public place compared to that of being infected by contact with an infectious individual in households ($\rho$) | 0–1 | 0.60 | 0.58–0.62 |
| The reduction in daily transmission probability by contact with an asymptomatic/pre-symptomatic infected individuals ($\varepsilon$) | 0–1 | 0.275 | 0.23–0.42 |
| The proportion of asymptomatic infections among newly infected individuals ($\psi$) | 0.101–0.23 (Byambasuren et al., 2020; He et al., 2021) | 0.174 | 0.101–0.23 |
| The mean incubation time (days) ($1/v$) | 5.0–6.7 (McAloon et al., 2020) | 5.8 | 5.0–6.7 |
| The interval from symptom onset to isolation in hospital or quarantine (days) ($1/q$) | 2–8 (Ng et al., 2020) | 4 | 2–8 |
| The interval from testing to diagnosis (days) ($1/g$) | 1–3 | 2 | 1–2 |
| The mean time from infection to recovery for asymptomatic infected individuals (days) ($1/r_{1}$) | 11–26 (Pan et al., 2020) | 22 | 11–26 |
| The mean time from diagnosis to recovery for symptomatic infected individuals (days) ($1/r_{2}$) | 11–26 (Pan et al., 2020) | 17 | 11–26 |
| Average daily probability of death due to disease during treatment ($\mu$) | 0.001–0.005 (Shen et al., 2020) | 0.002 | 0.0015–0.0037 |

For illustrative purposes, we simulated the historical epidemic trends in Victoria based on the state’s total population using the 1000 ‘good-fitting’ sets. We yielded the number of daily confirmed cases, the number of daily unknown-source cases, and the cumulative number of deaths, respectively. The outputs were compared with the corresponding calibration targets, as shown in Figure S5.


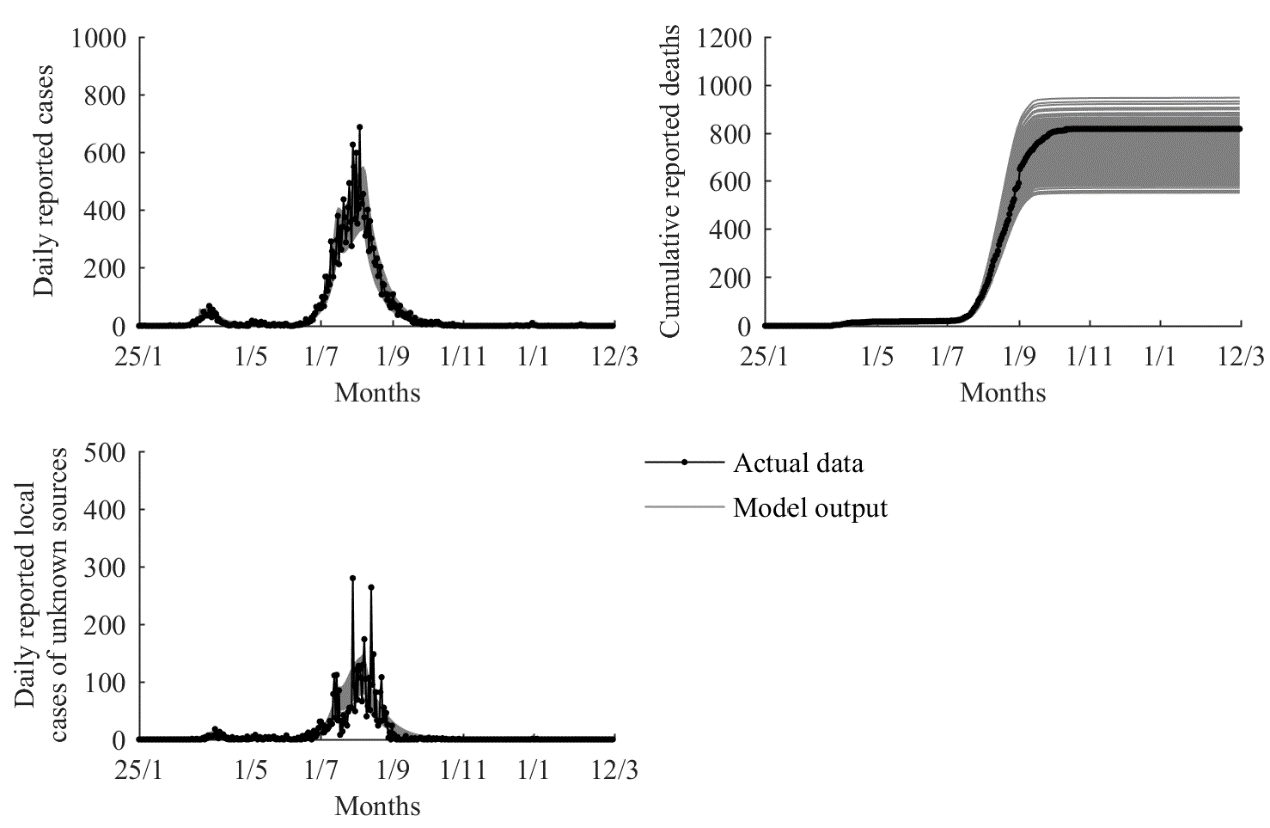


**Figure S5. Model outputs of COVID-19 outbreak trends in Victoria (25 January 2020 – 12 March 2021).**

1. **Estimation of undocumented cases**

Undocumented cases represented a potential risk of further community transmission of SARS-CoV-2. Three types of infections were considered ‘undocumented’ in our model. They were asymptomatic infections, pre-symptomatic infections, and symptomatic infections before diagnosis. The number of undocumented cases in historical outbreaks in Victoria was estimated based on the compartmental model and was shown in Figure S6. As can be seen, the cumulative number of cases rose at the fastest rate when the number of active undocumented cases reached a peak.


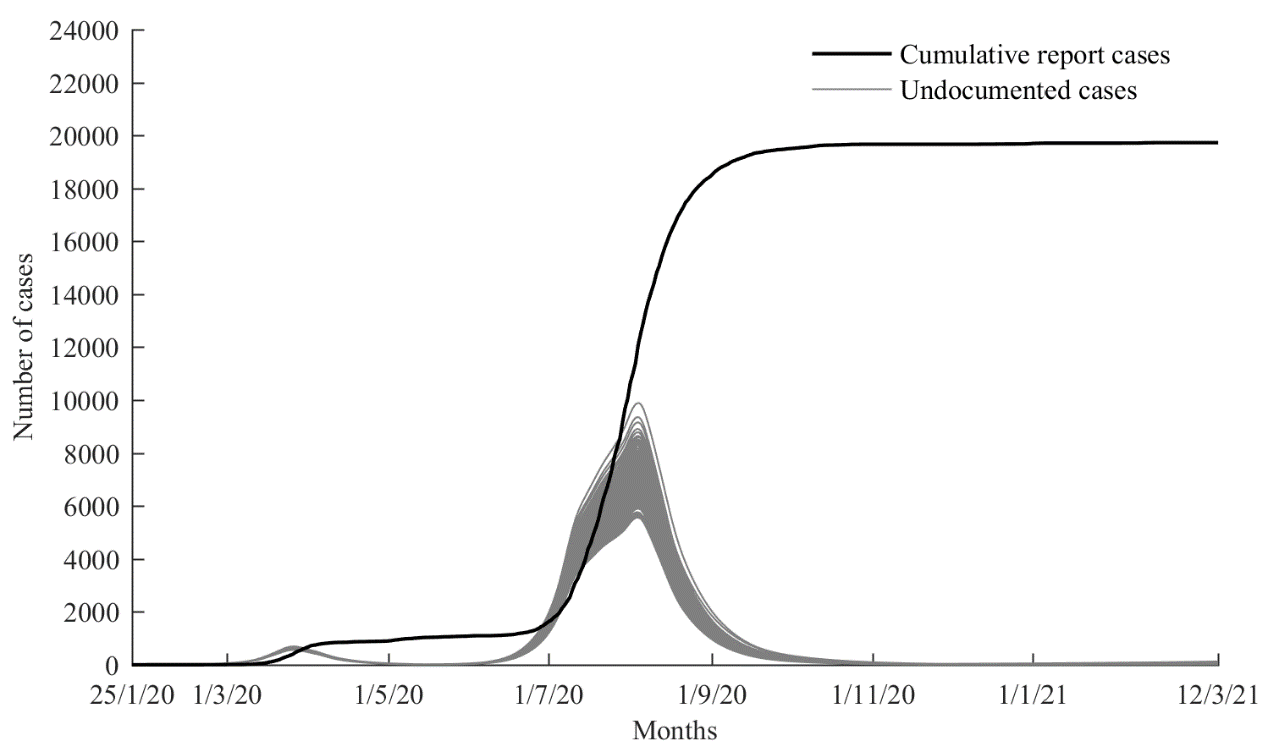


**Figure S6. Model outputs of undocumented cases (including asymptomatic infected individuals, pre-symptomatic infected individuals, and symptomatic infected individuals before diagnosis) in historical outbreaks in Victoria (25 January 2020 – 12 March 2021).**

1. **Association between the number of undocumented cases and the number of daily reported cases**

When a COVID-19 case was diagnosed, contact-tracers would adopt a backwards-tracing strategy to identify the source of transmission. A diagnosed case could be linked to the source of infection if (1) the source was symptomatic and had been diagnosed, or (2) the source was pre-symptomatic/asymptomatic but had been diagnosed through voluntary testing, or (3) the source was pre-symptomatic and was diagnosed due to the onset of symptoms during contact tracing. A diagnosed individual could have an unknown source of infection only when the source was asymptomatic and had not been detected by voluntary testing. Figure S7 demonstrated the potential reasons for cases being identified as unknown sources. With these assumptions, the actual reported number of unknown-source cases might reflect the number of potentially asymptomatic infections. In contrast, the number of known-source cases might reflect the number of pre-symptomatic/symptomatic infections in the population.


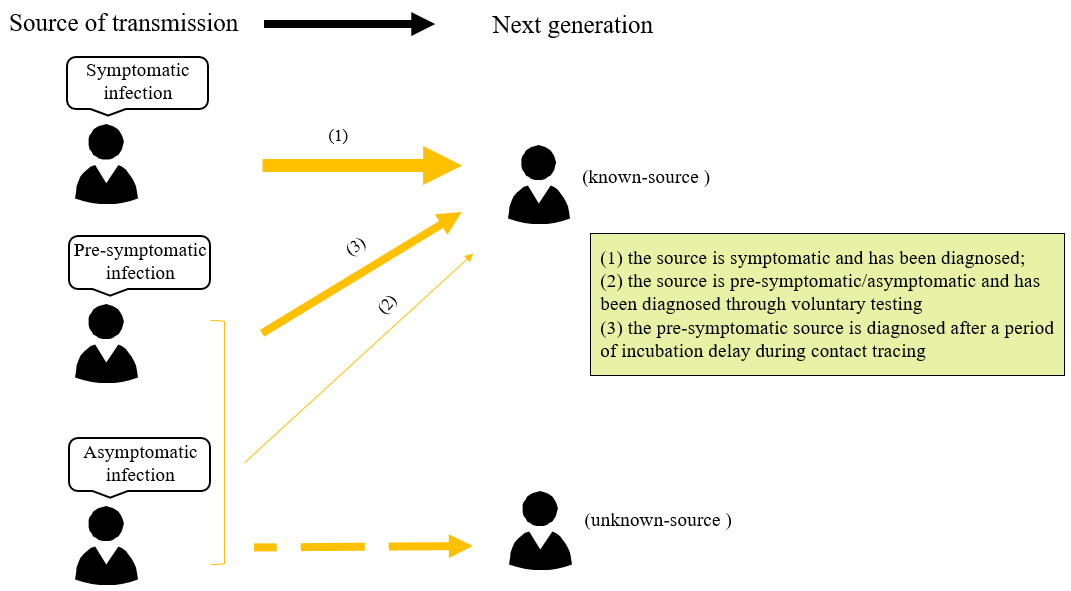


**Figure S7. The reasoning process for the source of confirmed cases.**

To quantify the above relationships, we explored the association between reported daily locally acquired cases and model-estimated potential undocumented infections using data from the second outbreak in Victoria (1 June 2020 – 30 October 2020). Data from this period were selected for analysis because (1) since all international passenger flights were cancelled in Victoria during the second outbreak, this largely excluded the impact of cases from overseas, and (2) this was the most severe of the COVID-19 outbreaks in Australia and data were adequate. An approximately linear relationship was found between the number of daily unknown-source cases and the number of estimated active asymptomatic infections with the Pearson correlation coefficient of 0.741 (linear regression, b = 13.32 [IQR: 12.9–14.14], p < 0.001). A significant linear relationship was found between the number of daily known-source cases and the number of estimated active pre-symptomatic/symptomatic infections with a Pearson correlation coefficient of 0.92 (linear regression, b = 13.13 [IQR: 12.96–13.9], p<0.001) (Figure S8–9).


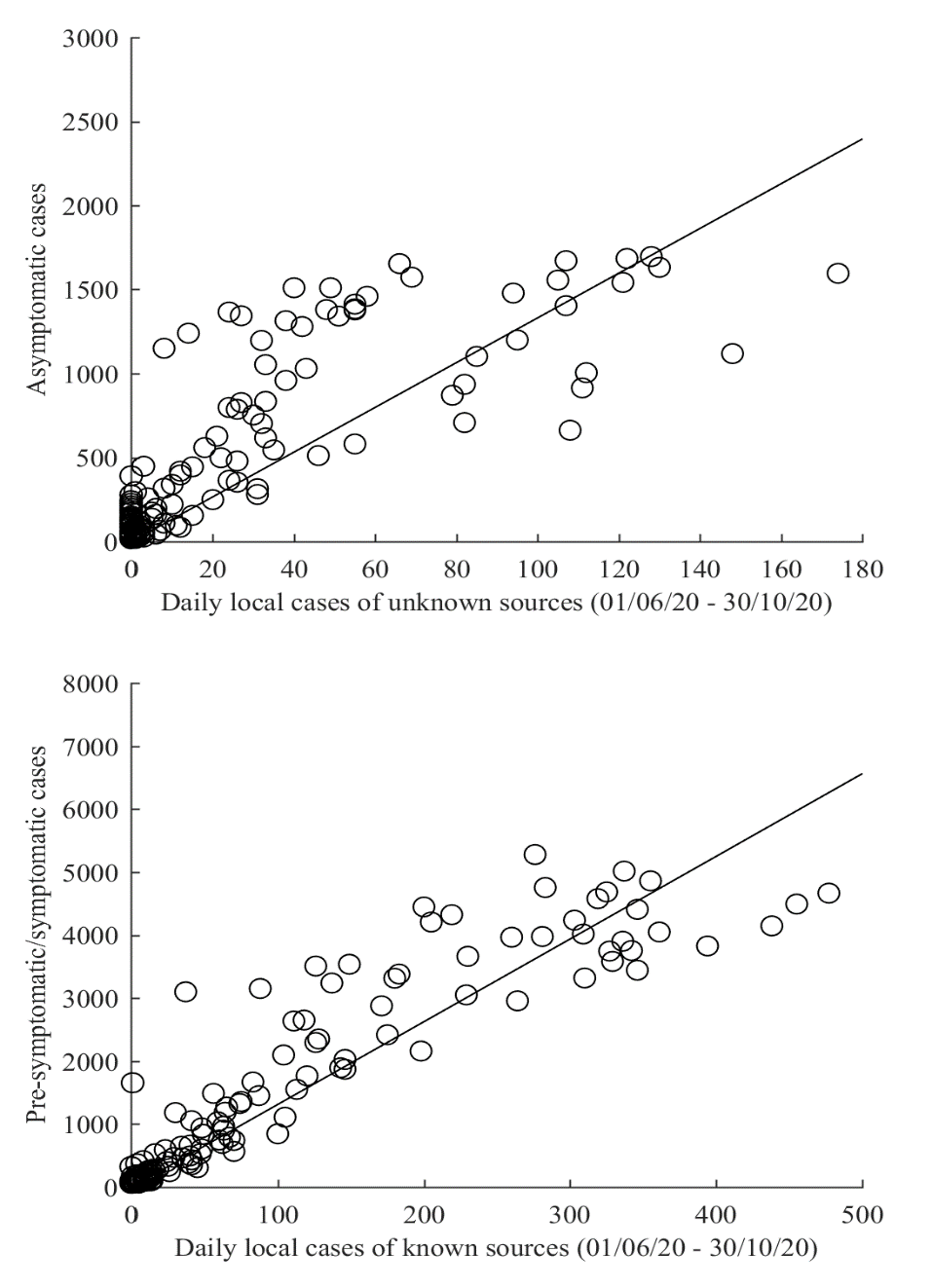


**Figure S8. The linear relationships between the number of daily unknown-source cases and the number of model-estimated asymptomatic infections, and between the number of daily known-source cases and the number of model-estimated pre-symptomatic/symptomatic infections.**


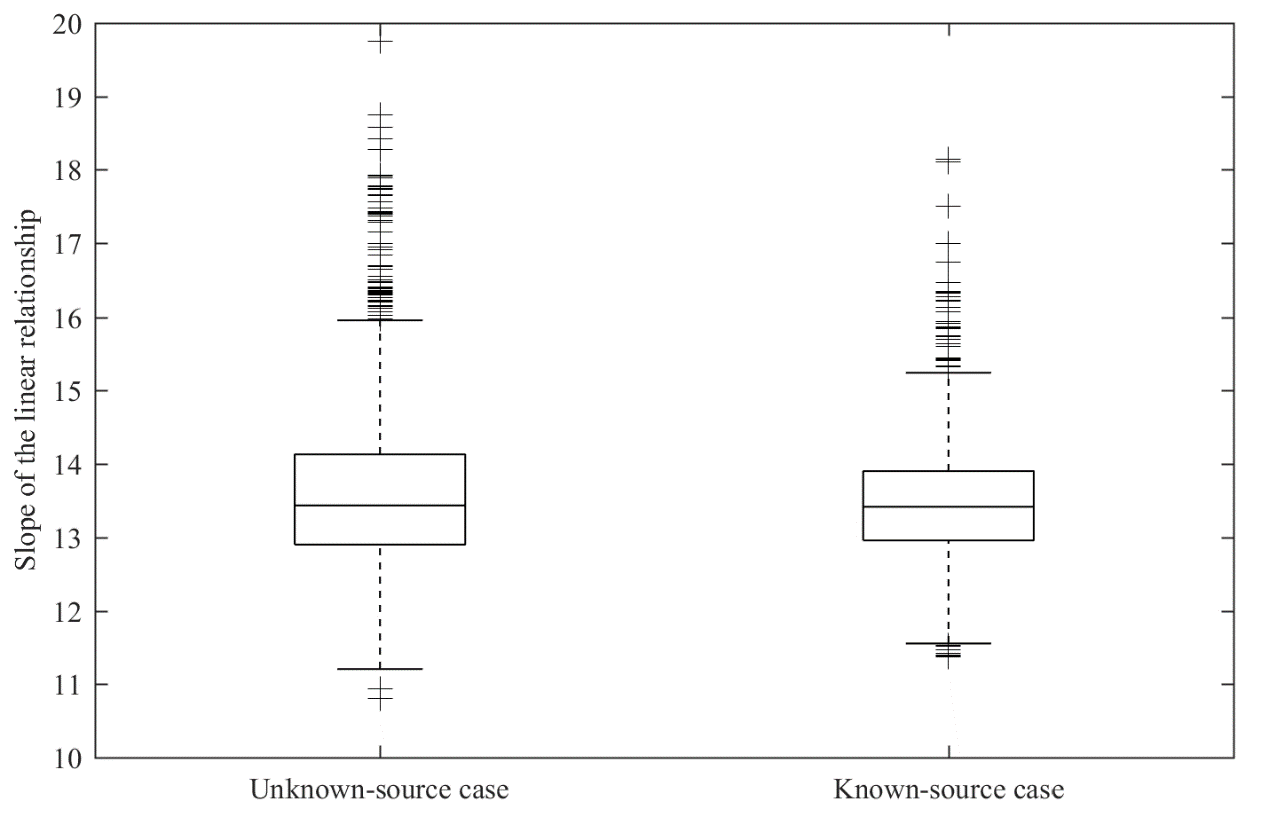


**Figure S9. Sensitivity analysis for the linear relationships.**

1. **Calculation of effective reproduction number**

The effective reproduction number (R_e_), the average number of secondary infections caused by a single infective at a given susceptible fraction, is calculated as the largest eigenvalue of the next generation matrix K = F×V^−1^ (Cintrón-Arias et al., 2009; Diekmann et al., 1990; Yang, 2020), where

$F$=

$$\left[ \begin{matrix} \left( \beta_{EA}^{fam}\cdot c_{f}+\beta_{EA}^{pub}\cdot c_{p}(t) \right)\cdot\psi& \left( \beta_{EA}^{fam}\cdot c_{f}+\beta_{EA}^{pub}\cdot c_{p}(t) \right)\cdot\psi& \left( \beta_{I}^{fam}\cdot c_{f}+\beta_{I}^{pub}\cdot c_{p}(t) \right)\cdot\psi\\ \left( \beta_{EA}^{fam}\cdot c_{f}+\beta_{EA}^{pub}\cdot c_{p}(t) \right)\cdot\left( 1-\psi\right) & \left( \beta_{EA}^{fam}\cdot c_{f}+\beta_{EA}^{pub}\cdot c_{p}(t) \right)\cdot\left( 1-\psi\right) & \left( \beta_{I}^{fam}\cdot c_{f}+\beta_{I}^{pub}\cdot c_{p}(t) \right)\cdot\left( 1-\psi\right) \\ 0 & 0 & 0 \end{matrix} \right]$$

and

$V$=

$$\left[ \begin{matrix} {(r}_{1}+\tau)+\left( \begin{aligned} \beta_{EA}^{fam}\cdot\xi\cdot g\cdot\left( c_{f}-1 \right)+ \\ \beta_{EA}^{pub}\cdot\xi\cdot g\cdot c_{p}(t) \end{aligned} \right)\cdot\psi\cdot\xi& \left( \begin{aligned} \beta_{EA}^{fam}\cdot\xi\cdot g\cdot\left( c_{f}-1 \right)+ \\ \beta_{EA}^{pub}\cdot\xi\cdot g\cdot c_{p}\left( t \right) \end{aligned} \right)\cdot\psi\cdot\xi& \left( \begin{aligned} \beta_{I}^{fam}\cdot q\cdot\left( c_{f}-1 \right)+ \\ \beta_{I}^{pub}\cdot q\cdot c_{p}(t) \end{aligned} \right)\cdot\psi\cdot\xi\\ \left( \begin{aligned} \beta_{EA}^{fam}\cdot\xi\cdot g\cdot\left( c_{f}-1 \right)+ \\ \beta_{EA}^{pub}\cdot\xi\cdot g\cdot c_{p}(t) \end{aligned} \right)\cdot(1-\psi)\cdot\xi& \left( v+\tau\right)+\left( \begin{aligned} \beta_{EA}^{fam}\cdot\xi\cdot g\cdot\left( c_{f}-1 \right)+ \\ \beta_{EA}^{pub}\cdot\xi\cdot g\cdot c_{p}(t) \end{aligned} \right)\cdot(1-\psi)\cdot\xi& \left( \begin{aligned} \beta_{I}^{fam}\cdot q\cdot\left( c_{f}-1 \right)+ \\ \beta_{I}^{pub}\cdot q\cdot c_{p}(t) \end{aligned} \right)\cdot(1-\psi)\cdot\xi\\ 0 & -v & q \end{matrix} \right]$$

The effective reproduction number under vaccination R_v_ is the number of secondary cases caused by one primary case introduced into a certain proportion of the vaccinated population (Farrington et al., 2003; Scherer and McLean, 2002). We assumed that individuals who receive the vaccine and develop an immune response would no longer be infected over a period of time. Thus, R_v_ was expressed as:

|  | $R_{V}=(1-\theta\cdot p)\cdot R_{e}$ | (7) |
| --- | --- | --- |

Where $\theta$ denotes the efficacy of the vaccine and $p$ denotes the vaccination coverage.

1. **Estimation of the average infectious period**

Based on the definition of R_e_, i.e., the number of secondary cases generated by a single infectious case, we multiplied R_e_ with the number of undocumented cases to obtain the total number of secondary cases caused by the current source of infection during the average infectious period.

We estimated the average infectious period for individuals infected with SARS-COV-2 as follows. Previous studies have indicated that the mean incubation period for pre-symptomatic infected individuals (who later became symptomatic) was approximately 5.8 (95% CI 5.0–6.7) days (McAloon et al., 2020), and the interval from symptom onset to isolation in hospital was about 5.6 (IQR 2–8) days (Ng et al., 2020). Therefore, the average infectious period for a pre-symptomatic case was approximately 11.4 days. In contrast, asymptomatic infected individuals who never presented any symptoms would additionally experience an asymptomatic recovery period of 17±4 (range 11–26) days (Pan et al., 2020). We assumed that the interval between infection and onset of recovery for asymptomatic infected individuals was equivalent to the mean incubation period of symptomatic infected individuals. Therefore, the overall infectious period (i.e. incubation period plus asymptomatic recovery period) for an asymptomatic case was about 22.8 days. Given the proportion of asymptomatic cases among infected cases is 17% (95% CI 14–20%) (He et al., 2021), the weighted average infectious period of a SARS-COV-2 infected individual was estimated to be about 14 days.

1. **Calculation of critical timing for intervention commencement**

We defined the number of reported cases that would trigger intervention commencement to reduce Re to below one and maintain the average number of daily reported cases over a 7-day period into interventions to be the critical timing for intervention implementation.

We assumed that Re would decrease uniformly from its initial value to one after 7 days into the interventions. We used an autoregressive approach to estimate the average number of reported cases 7 days into the intervention. First, we projected the average daily number of cases over the next 7 days using the number of reported locally acquired cases and Re on the day of intervention commencement. We assumed that the average daily number of cases equals the number of cases on the 4th day; we then linearly interpolated the cases to obtain the number of reported cases one day after intervention. We used a similar approach iteratively to estimate the number of reported cases over the first 7 days after interventions. The critical timing was obtained by limiting the estimated average daily number of cases over the next 7 days after interventions to an average of ≤10 cases/day.

1. **Model validation**

To validate the reliability and generalisability of the model predictions, we compared the projected number of cases over the next 7 days with the actual number of reported cases over a 7-day period in each of Victoria, New South Wales, Australian Capital Territory, and Western Australia, for the period 25 January 2020 –12 March 2021(Figure S10). It can be seen that the model predictions matched well with the actual epidemic trends, with an R-squared of 0.99, 0.88, 0.77, and 0.88, respectively.


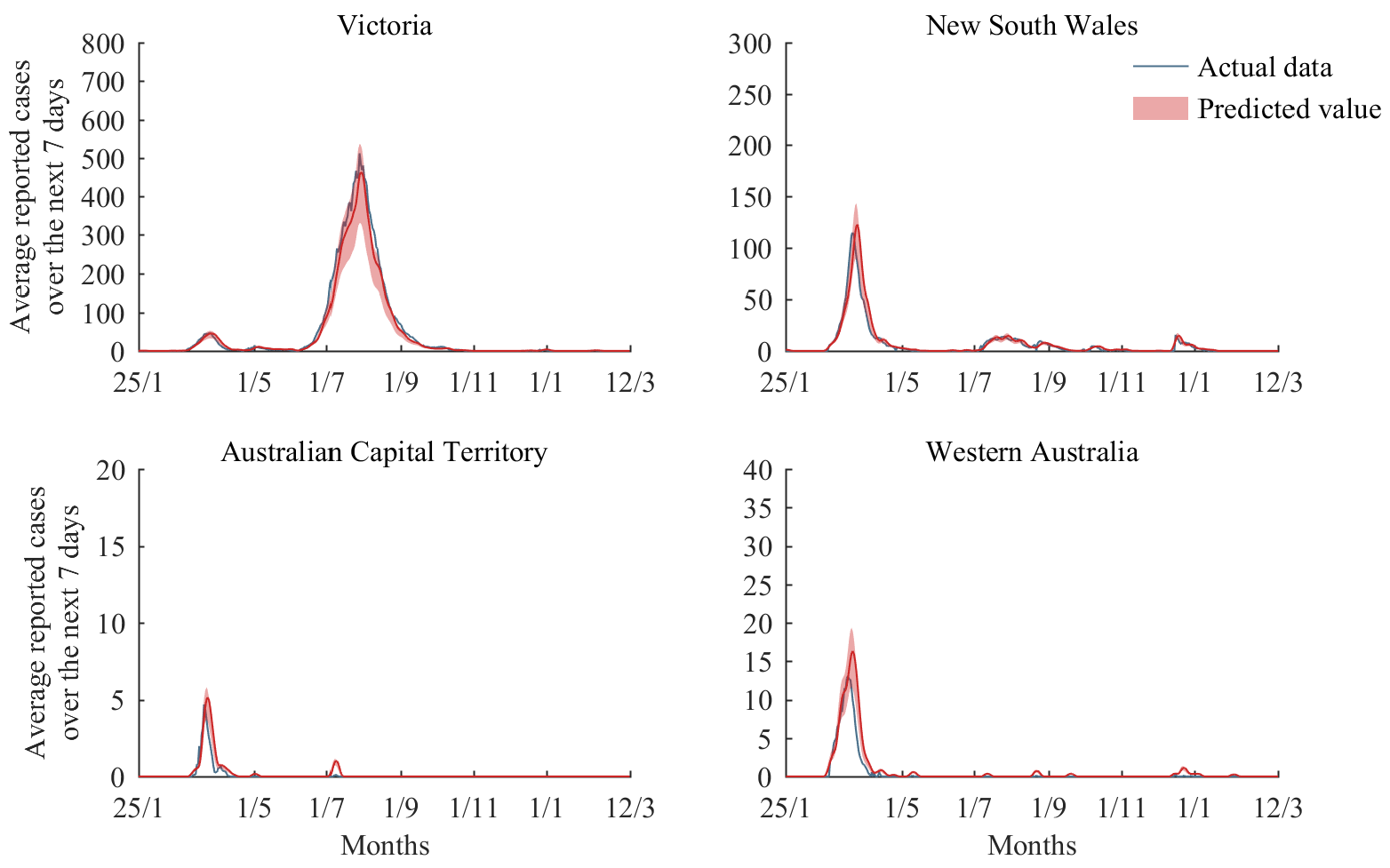


**Figure S10. Model predictions of COVID-19 outbreak trends in Victoria, New South Wales, the Australian Capital Territory, and Western Australia (25 January 2020 –12 March 2021).**

1. **Additions to the results**

Figure S11 and Figure S12 demonstrated the effect of various levels of reduction in social activity and face mask coverage on Re under different scenarios when the effectiveness of the mask was at the lower limit (50%) and the upper limit (95%). Figure S13 demonstrated the effect of various levels of reduction in social activity and face mask coverage on Re in mixed epidemics when the vaccination coverage reaches the target of 70%.


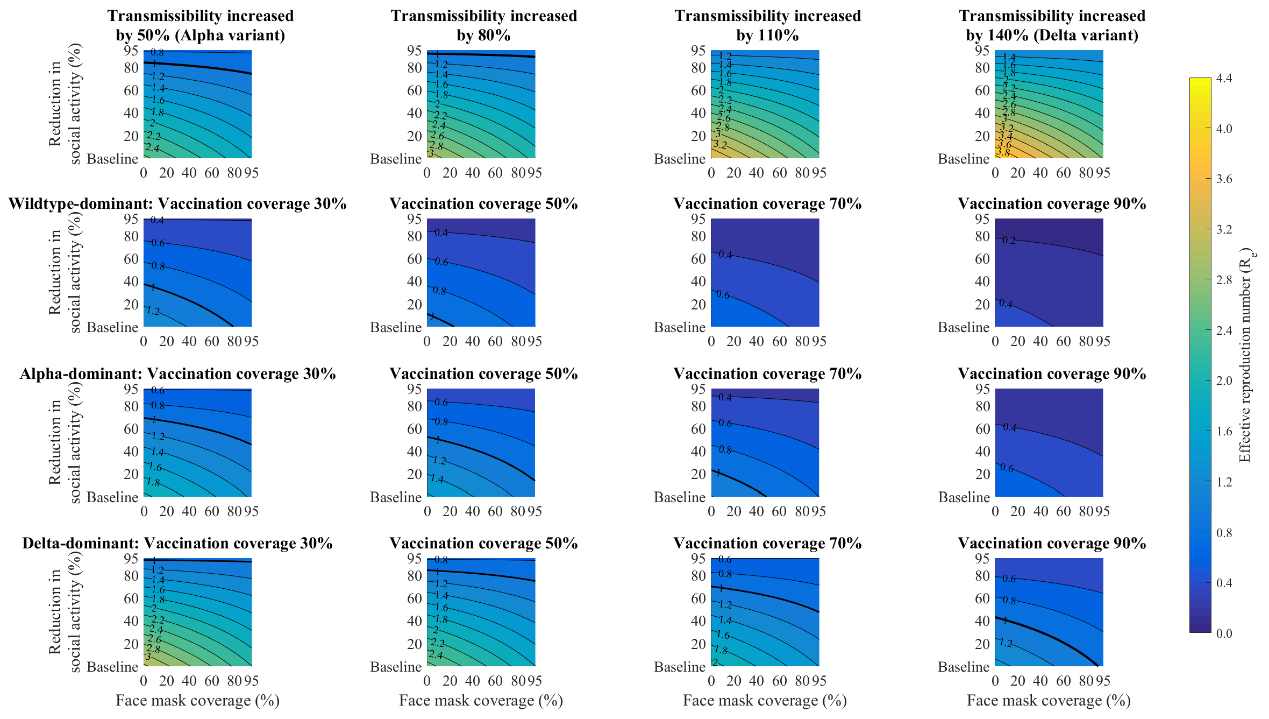


**Figure S11 Effect of various levels of reduction in social activity and face mask coverage on Re under different scenarios when the effectiveness of face mask was at the lower limit (50%).**

Abbreviations: Re, effective reproduction number.


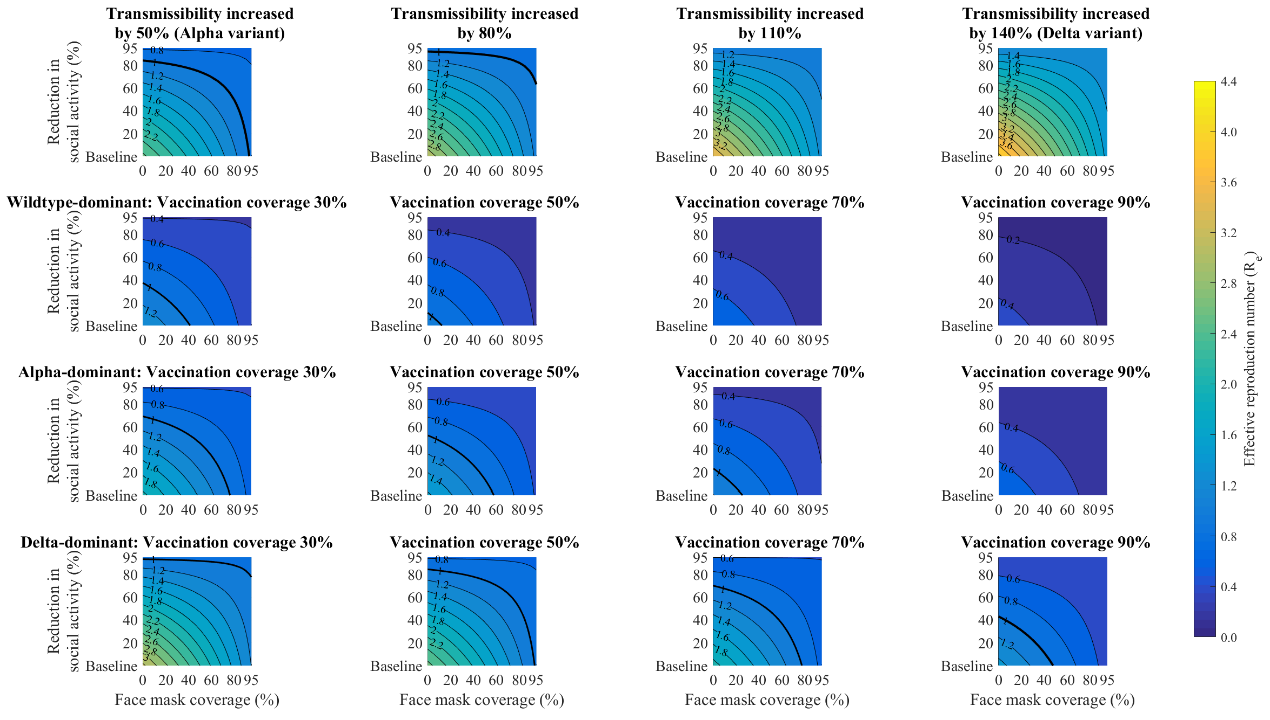


**Figure S12 Effect of various levels of reduction in social activity and face mask coverage on Re under different scenarios when the effectiveness of face mask was at the upper limit (95%).**

Abbreviations: Re, effective reproduction number.


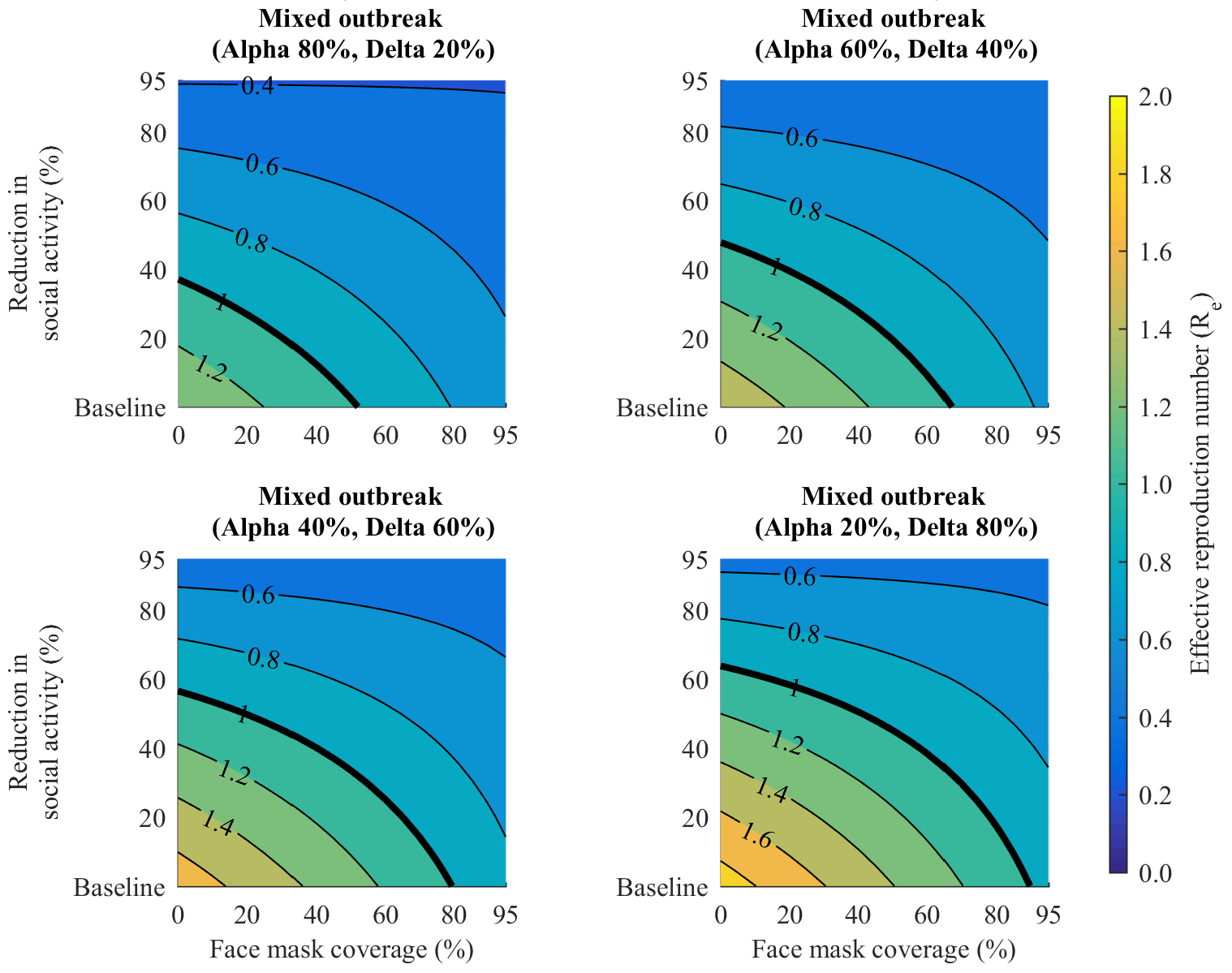


**Figure S13 Effect of various levels of reduction in social activity and face mask coverage on Re in mixed epidemics when the vaccination coverage reaches the target of 70%.**

Abbreviations: Re, effective reproduction number.

**References:**

Alsubaie H, Goldenberg M, Grantcharov T. Quantifying recall bias in surgical safety: a need for a modern approach to morbidity and mortality reviews. Can J Surg 2019;62(1):39–43, doi:10.1503/cjs.017317.

Australian Government Department of Health. Australia's vaccine agreements. <https://www.health.gov.au/node/18777/australias-vaccine-agreements>, 2021 (accessed 4 Nov 2021).

Baden LR, El Sahly HM, Essink B, Kotloff K, Frey S, Novak R, et al. Efficacy and safety of the mRNA-1273 SARS-CoV-2 vaccine. N Engl J Med 2021;384(5):403–16, doi:10.1056/NEJMoa2035389.

Byambasuren O, Cardona M, Bell K, Clark J, McLaws ML, Glasziou P. Estimating the extent of true asymptomatic COVID-19 and its potential for community transmission: systematic review and meta-analysis. JAMMI 2020;5(4):223–34, doi:10.3138/jammi-2020-0030.

Centers for Disease Control and Prevention. Case investigation & contact tracing guidance: contact tracing for COVID-19. <https://www.cdc.gov/coronavirus/2019-ncov/php/contact-tracing/contact-tracing-plan/contact-tracing.html>, 2021 (accessed 4 Nov 2021).

Chu DK, Akl EA, Duda S, Solo K, Yaacoub S, Schünemann HJ, et al. Physical distancing, face masks, and eye protection to prevent person-to-person transmission of SARS-CoV-2 and COVID-19: a systematic review and meta-analysis. Lancet 2020;395(10242):1973–87, doi:10.1016/S0140-6736(20)31142-9.

Cintrón-Arias A, Castillo-Chávez C, Bettencourt LM, Lloyd AL, Banks HT. The estimation of the effective reproductive number from disease outbreak data. Math Biosci Eng 2009;6(2):261–82, doi:10.3934/mbe.2009.6.261.

Diekmann O, Heesterbeek JA, Metz JA. On the definition and the computation of the basic reproduction ratio R_0_ in models for infectious diseases in heterogeneous populations. J Math Biol 1990;28(4):365–82, doi:10.1007/BF00178324.

Dyani L. Where COVID contact-tracing went wrong. Nature 2020;588:384–8. <https://media.nature.com/original/magazine-assets/d41586-020-03518-4/d41586-020-03518-4.pdf>.

Farrington CP. On vaccine efficacy and reproduction numbers. Math Biosci 2003;185(1):89–109, doi:10.1016/s0025-5564(03)00061-0.

Fitzhugh Mullan Institute for Health Workforce Equity, the George Washington University. Contact tracing workforce estimator. <https://www.gwhwi.org/estimator-613404.html>, 2020 (accessed 4 Nov 2021).

Google. COVID-19 Community Mobility Reports. <https://www.google.com/covid19/mobility>, 2021 (accessed 4 Nov 2021).

He J, Guo Y, Mao R, Zhang J. Proportion of asymptomatic coronavirus disease 2019: A systematic review and meta-analysis. J Med Virol 2021;93(2):820–30, doi:10.1002/jmv.26326.

Howard J, Huang A, Li Z, Tufekci Z, Zdimal V, van der Westhuizen HM, et al. An evidence review of face masks against COVID-19. Proc Natl Acad Sci U S A 2021;118(4):e2014564118, doi:10.1073/pnas.2014564118.

Institute for Health Metrics and Evaluation. COVID-19 Projections-Australia. <https://covid19.healthdata.org/australia?view=cumulative-deaths&tab=trend>, 2021 (accessed 4 Nov 2021).

Integrated Disease Surveillance Programme National Centre for Disease Control. Guidelines for contact tracing of COVID-19 cases in community settings. <https://ncdc.gov.in/WriteReadData/l892s/5543723831596613278.pdf>, 2020 (accessed 4 Nov 2021).

Kim JJ, Kuntz KM, Stout NK, Mahmud S, Villa LL, Franco EL, et al. Multiparameter calibration of a natural history model of cervical cancer. Am J Epidemiol 2007;166(2):137–50, doi:10.1093/aje/kwm086.

Lopez Bernal J, Andrews N, Gower C, Gallagher E, Simmons R, Thelwall S, et al. Effectiveness of Covid-19 Vaccines against the B.1.617.2 (Delta) Variant. N Engl J Med 2021;385(7):585–94, doi:10.1056/NEJMoa2108891.

MacIntyre CR, Dwyer D, Seale H, Fasher M, Booy R, Cheung P, et al. The first randomized, controlled clinical trial of mask use in households to prevent respiratory virus transmission. Int J Infect Dis 2008;12:e328, doi:10.1016/j.ijid.2008.05.877.

McAloon C, Collins Á, Hunt K, Barber A, Byrne AW, Butler F, et al. Incubation period of COVID-19: a rapid systematic review and meta-analysis of observational research. BMJ Open 2020;10(8): e039652, doi:10.1136/bmjopen-2020-039652.

McCall J. Genetic algorithms for modelling and optimisation. J Comput Appl Math 2005;184(1): 205–22, doi:10.1016/j.cam.2004.07.034.

Ng Y, Li Z, Chua YX, Chaw WL, Zhao Z, Er B, et al. Evaluation of the effectiveness of surveillance and containment measures for the first 100 patients with COVID-19 in Singapore - January 2-February 29, 2020. MMWR Morb Mortal Wkly Rep 2020;69(11):307–11, doi:10.15585/mmwr.mm6911e1.

Pan F, Ye T, Sun P, Gui S, Liang B, Li L, et al. Time course of lung changes at chest CT during recovery from Coronavirus Disease 2019 (COVID-19). Radiology 2020;295(3):715–21, doi:10.1148/radiol.2020200370.

Polack FP, Thomas SJ, Kitchin N, Absalon J, Gurtman A, Lockhart S, et al. Safety and efficacy of the BNT162b2 mRNA Covid-19 vaccine. N Engl J Med 2020;383(27):2603–15, doi:10.1056/NEJMoa2034577.

Scherer A, McLean A. Mathematical models of vaccination. Br Med Bull 2002;62(1):187–99, doi:10.1093/bmb/62.1.187.

Sheikh A, McMenamin J, Taylor B, Robertson C; Public Health Scotland and the EAVE II Collaborators. SARS-CoV-2 Delta VOC in Scotland: demographics, risk of hospital admission, and vaccine effectiveness. Lancet 2021;397(10293):2461–2, doi:10.1016/S0140-6736(21)01358-1.

Shen M, Peng Z, Guo Y, Rong L, Li Y, Xiao Y, et al. Assessing the effects of metropolitan-wide quarantine on the spread of COVID-19 in public space and households. Int J Infect Dis 2020;96:503–5, doi:10.1016/j.ijid.2020.05.019.

Stowe J, Andrews N, Gower C, Gallagher E, Utsi L, Simmons R, et al. Effectiveness of COVID-19 vaccines against hospital admission with the Delta variant. PHE national. <https://khub.net/web/phe-national/public-library/-/document_library/v2WsRK3ZlEig/view/479607266>, 2021 (accessed 4 Nov 2021).

Voysey M, Clemens SAC, Madhi SA, Weckx LY, Folegatti PM, Aley PK, et al. Safety and efficacy of the ChAdOx1 nCoV-19 vaccine (AZD1222) against SARS-CoV-2: an interim analysis of four randomized controlled trials in Brazil, South Africa, and the UK. Lancet 2021;397(10269):99–111, doi:10.1016/S0140-6736(20)32661-1.

Yang W. Transmission dynamics of and insights from the 2018-2019 measles outbreak in New York City: A modeling study. Sci Adv 2020;6(22):eaaz4037, doi:10.1126/sciadv.aaz4037.

Zhang L, Shen M, Ma X, Su S, Gong W, Wang J, et al. What Is Required to Prevent a Second Major Outbreak of SARS-CoV-2 upon Lifting Quarantine in Wuhan City, China. Innovation (N Y) 2020;1(1):100006, doi:10.1016/j.xinn.2020.04.006.
